# Socio-economic predictors of environmental performance among African nations

**DOI:** 10.1038/s41598-019-45762-3

**Published:** 2019-06-26

**Authors:** Corey J. A. Bradshaw, Enrico Di Minin

**Affiliations:** 1 Global Ecology, College of Science and Engineering, Flinders University, GPO Box 2100, Adelaide, South Australia 5001 Australia; 2 Helsinki Lab of Interdisciplinary Conservation Science, Department of Geosciences and Geography, University of Helsinki, FI00014 Helsinki, Finland; 3 Helsinki Institute of Sustainability Science (HELSUS), University of Helsinki, FI00014 Helsinki, Finland; 4 School of Life Sciences, University of KwaZulu-Natal, Durban, 4000 South Africa

**Keywords:** Biodiversity, Environmental sciences

## Abstract

Socio-economic changes in Africa have increased pressure on the continent’s ecosystems. Most research investigating environmental change has focused on the changing status of specific species or communities and protected areas, but has largely neglected the broad-scale socio-economic conditions underlying environmental degradation. We tested national-scale hypotheses regarding the socio-economic predictors of ecosystem change and degradation across Africa, hypothesizing that human density and economic development increase the likelihood of cumulative environmental damage. Our combined environmental performance rank includes national ecological footprint, proportional species threat, recent deforestation, freshwater removal, livestock density, cropland coverage, and per capita emissions. Countries like Central African Republic, Botswana, Namibia, and Congo have the best relative environmental performance overall. Structural equation models indicate that increasing population density and overall economic activity (per capita gross domestic product corrected for purchasing-power parity) are the most strongly correlated with greater environmental degradation, while greater wealth inequality (Gini index) correlates with better environmental performance. This represents the first Africa-scale assessment of the socio-economic correlates of environmental degradation, and suggests that dedicated family planning to reduce population growth, and economic development that limits agricultural expansion (cf. intensification) are needed to support environmental sustainability.

## Introduction

Africa is the only continent to have passed through the late-Pleistocene/early-Holocene megafauna extinction pulse relatively intact compared to most other continents^1^, although many megafauna extinctions still occurred there in the mid-Pleistocene^2^ and later^3^. This means that today, Africa is the last global refugium of a functionally intact assemblage of large herbivores, carnivores, and scavengers^4^, and it boasts the world’s highest mammal alpha diversity^5^. However, although much of the African continent experienced some later environmental change during its colonial period^6^, many of its natural resources have only recently (i.e., last few decades) been exposed to broad-scale exploitation compared to other regions of the world^7,8^. In addition, the African continent has the greatest projected growth in human population size over the next century, and is set to achieve some of the world’s highest human densities by 2100^9^ — the entire continent is home to over 1.2 billion people today and is projected to increase by between five- and seven-fold to nearly six billion by the end of the century based on current fertility rates^9^. Further, Africa’s relatively slow rate of fertility decline (about one third of Latin America’s and Asia’s trajectories since the 1950s), which in some countries is even stabilizing^10^, means that there is little prospect to avoid this projected growth in the human population.

Globally, ongoing species extinctions, the rising threat risk across all major taxonomic groups, and the declining abundance of biodiversity globally^11^ — over the last half century in particular^12^ — have arisen primarily from massive human modification of the biosphere^13^. The principal drivers of these population and species extinctions are clear^14^: habitat conversion^15^ — much of it from agricultural expansion^7^, road development^16^, over-exploitation^15^, pollution^17^, urbanization^18,19^, climate disruption^20^, and the synergies among these^21^. But these proximate drivers sometimes belie the ultimate driver of them all — human population expansion (both numerical and geographical) and the consumption of Earth’s resources this implies^9^. Indeed globally, human population density predicts the number of threatened species among nations^22–24^, so the inexorable growth of the global human population from 7.5 billion to possibly over 12 billion by the end of the century^9^ will undoubtedly exacerbate the extinction risk of many species.

However, the direct evidence for the negative effects of human population size, density, and growth on biodiversity is often equivocal, or at least confounded with other conditions. For example, there is only a weak correlation globally between human population density and species extinctions because of the spatial congruence between human population size and species richness, a lack of data on extinctions, and the variability across methods^25^. However, there is evidence that current human population densities and growth rates are higher in Biodiversity Hotspots (i.e., where the greatest potential species loss occurs) than elsewhere^26,27^, and there is also a positive historical relationship between human population size and threats to biodiversity at national scales^28,29^. While the highest recent mammal extinctions in Africa overall did not correspond with the highest human population densities, the absolute number of extinctions in southern Africa coincided with high human population densities^30^. In Europe, there is a century-scale time lag between increasing human population density and current biodiversity threat^31^. Furthermore, 50% of tropical protected areas are experiencing biodiversity loss because of high human population growth and locally or foreign-driven consumption at their edges^32^. For these same protected areas, human population size is also negatively correlated with a protected area’s biodiversity ‘health’^33^.

Of course, human population size is only part of the equation with respect to predicting environmental degradation, because consumption rates add to and interact with population size^34^, albeit in different ways depending on the wealth and culture of the human population in question^35,36^. Indeed, previous work suggests that variation in environmental degradation at a national scale is best described by a country’s accumulated ‘wealth’ as measured by gross measures of economic turnover (e.g., gross national income and gross domestic product), with a country’s population size inextricably linked to the magnitude of its economy^37^. However, that global study perhaps unjustly penalized those countries where most broad-scale environmental degradation had happened recently^37^. Therefore, a more regional analysis of national environmental performance focussing on African countries with more similar histories, cultures, economies, and ecosystems might provide additional insights into the relationships and interactions among economics, human population trends, and the overall state of a nation’s environment.

Given increasing exploitation and the rapid growth of its human population, Africa stands to lose many more of its already threatened species and ecosystems over the coming decades, especially as legal and illegal overseas demand (especially from China) for timber, minerals, fuels, agricultural, seafood, and wildlife products increases^7,8,38,39^. Civil unrest^40^, a recent history of poor governance and corruption in many states^41^, a rising prevalence of organized crime^42,43^, and extensive (but diminishing) poverty^44^ threaten to exacerbate Africa’s environmental situation further. But which African states are doing better than their peers in environmental custodianship and performance? As pressures on the environment grow, a quantitative index of relative environmental performance among African nations is now needed to quantify the relative contribution the socio-economic, demographic, and industrial drivers of environmental degradation, and more importantly, to highlight which countries have performed better at resisting the loss of their biodiversity.

In this paper, we combine several national-scale metrics of environmental performance (ecological footprint, megafauna conservation index, species threat, freshwater removals, forest loss, livestock density, cropland extent, greenhouse-gas emissions) for mainland Africa countries (including Madagascar, but excluding small-island nations because the latter tend to be outliers for metrics expressed per area or per capita) to construct a relative environmental performance indicator specific to Africa. This approach avoids the unfair comparison of environmental performance in African nations against non-African states. We then examine the correlation between environmental rank and the following socio-economic indicators as possible explanatory drivers for the variation observed using structural equation models: (*i*) human population density, (*ii*) wealth (gross domestic product), (*iii*) wealth distribution (Gini index), (*iv*) governance quality, and (*v*) commitment to environmental protection (through the establishment of dedicated protected areas). Our hypotheses are that environmental performance of a nation declines as its population density^25^, wealth^37^, and wealth disparity^45^ increase, and as its governance quality^46,47^ and area under protection^33^ declines.

## Results

**Figure 1:**
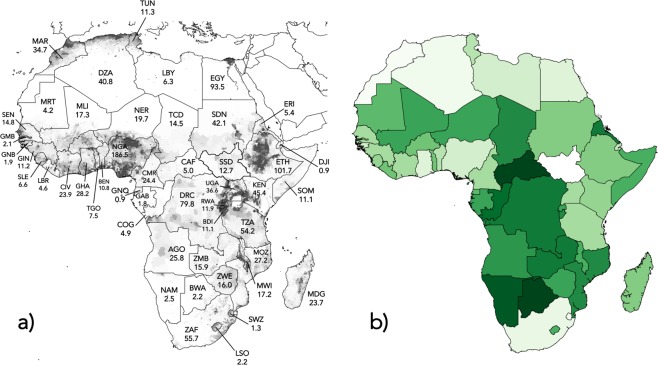
(**a**) Map of countries in Africa with background shading indicating approximate relative density of human populations (data from the Global Rural-Urban Mapping Project GRUMP V1; http://sedac.ciesin.columbia.edu/data/collection/grump-v1/methods). Each country (3-letter ISO country codes given in Table 2) is also shown with its approximate mid-2016 total human population size (Population Reference Bureau; www.prb.org) in millions. (**b**) African countries shaded according to relative environmental performance (darker green indicates better relative environmental performance; see Table 2 for values).

A non-parametric (Kendall’s *τ*) correlation matrix among the component environmental metrics demonstrated only weak or moderate (most *τ* ≤ |0.385|) relationships among variables (Table 1), so we elected to keep all hypothesized correlates in the saturated (i.e., including all hypothesized correlates) structural equation model. However, there was a reasonably strong correlation (−0.523) between freshwater removal and forest loss among countries — a lack of an obvious mechanistic link between the two suggests that neither can be excluded (Table 1). After calculating the geometric mean rank of countries for which there were at least seven component environmental variables, the top five countries for best environmental performance were (in order) Central African Republic, Botswana, Namibia, Congo, and Democratic Republic of Congo (Table 2; Fig. 1). The five worst environmental performers (in order of worst to less bad) were: Morocco, Algeria, Swaziland, South Africa, and Ghana (Table 2).

**Table 1:** Correlation (Kendall’s *τ*) matrix of environmental component variable ranks.

	EF	MCI	THR	FWR	FRL	LVS	CPL
MCI	0.341						
THR	0.159	0.249					
FWR	0.336	0.190	0.238				
FRL	−0.356	−0.246	−0.064	−0.523			
LVS	−0.145	−0.066	0.050	0.076	−0.132		
CPL	−0.110	−0.036	0.244	−0.108	0.303	0.276	
EMI	0.385	0.131	0.149	0.233	−0.187	−0.161	−0.018

**EF** = ecological footprint^48^; **MCI** = megafauna conservation index^49^; **THR** = relative species threat (number of IUCN Red List species classified as *Critically Endangered*, *Endangered*, *Vulnerable*, or *Near Threatened* divided by total number of species assessed; iucnredlist.org); **FWR** = freshwater removals (percent of internal resources; data.worldbank.org); **FRL** = recent (2000 to 2012) proportional forest loss^50^; **LVS** = livestock (cattle, pigs, buffaloes, sheep, and goats per hectare of arable land; fao.org/faostat); **CPL** = extent of permanent croplands (percent of total land area; data.worldbank.org); **EMI** = greenhouse-gas emissions (CO_2_-e per capita in 2013; data.worldbank.org).

**Table 2:** Ranking results (*n* = 48 countries) based on the composite environmental performance index (ENV_gm_ = geometric mean of the eight environmental component variable ranks). **ISO** = Alpha-3 country code; **EF** = ecological footprint^48^; **MCI** = megafauna conservation index^49^; **THR** = relative species threat (number of IUCN Red List species classified as *Critically Endangered*, *Endangered*, *Vulnerable*, or *Near Threatened* divided by total number of species assessed; iucnredlist.org); **FWR** = freshwater removals (percent of internal resources; data.worldbank.org); **FRL** = recent (2000 to 2012) proportional forest loss^50^; **LVS** = livestock (cattle, pigs, buffaloes, sheep, and goats per hectare of arable land; fao.org/faostat); **CPL** = extent of permanent croplands (percent of total land area; data.worldbank.org); **EMI** = greenhouse-gas emissions (CO_2_-e per capita in 2013; data.worldbank.org).

Country	ISO	environmental component variable ranks								ENV_gm_
		EF	MCI	THR	FWR	FRL	LVS	CPL	EMI	
Cent Afr Rep	CAF	18	5	1	2	25	44	11	6	7.754
Botswana	BWA	44	1	4	31	10	7	1	42	7.955
Namibia	NAM	NA	2	18	24	12	5	3	38	9.276
Congo	COG	20	20	9	1	27	2	14	31	9.790
Dem Rep Congo	COD	3	33	23	5	43	10	21	2	10.943
Eritrea	ERI	1	34	45	37	4	27	4	NA	11.363
Zambia	ZMB	8	6	8	18	39	11	7	21	12.020
Chad	TCD	31	23	11	26	19	13	5	3	12.876
Burundi	BDI	2	22	22	21	24	43	45	1	13.240
Mozambique	MOZ	5	10	38	12	46	4	20	15	13.724
Angola	AGO	6	17	12	10	38	7	15	39	14.454
Gabon	GAB	NA	30	17	6	23	3	24	44	15.581
Mali	MLI	30	29	5	34	16	30	10	5	15.623
Zimbabwe	ZWE	17	4	6	39	29	17	16	36	16.102
Rwanda	RWA	7	7	21	16	22	47	42	7	16.309
Niger	NER	32	26	15	38	6	24.5	9	9	16.557
Somalia	SOM	24	44	40	43	11	20	6	4	17.690
Lesotho	LSO	34	32	16	11	4	32	12	37	17.972
Malawi	MWI	4	9	37	32	33	35.5	30	8	18.190
Eq Guinea	GNQ	NA	28	20	4	34	7	32	46	18.650
Mauritania	MRT	42	46	27	46	7	15	2	33	18.950
Togo	TGO	13	12	10	15	35	33	36	26	19.970
Liberia	LBR	16	41	33	3	48	14	31	20	20.139
Burkina Faso	BFA	21	18	7	30	26	45	19	16	20.246
Madagascar	MDG	10	NA	50	25	41	12	27	13	21.550
Ethiopia	ETH	11	21	29	35	17	46	29	10	21.913
Sudan	SDN	28	31	35	47	9	NA	8	25	22.094
Kenya	KEN	12	8	41	36	20	38	26	23	22.444
Sierra Leone	SLE	23	25	30	7	44	21	33	18	22.525
Gambia	GMB	9	38	14	22	32	41.5	23	22	22.711
Swaziland	SWZ	39	27	3	42	28	26	25	35	23.220
Tanzania	TZA	27	3	48	28	42	31	34	19	23.454
Cameroon	CMR	15	16	39	9	30	40	38	24	23.474
Senegal	SEN	19	13	26	33	21	37	18	32	23.557
Côte d’Ivoire	CIV	22	14	32	19	49	9	46	27	23.789
Guinea	GIN	29	36	34	8	36	18	35	17	23.984
Uganda	UGA	26	11	31	17	37	39	43	12	24.058
Benin	BEN	25	15	13	14	47	35.5	39	30	24.580
Tunisia	TUN	40	37	42	45	1	28	47	43	25.115
Djibouti	DJI	43	45	47	29	4	24.5	NA	34	26.336
Guinea Bissau	GNB	33	43	19	13	45	34	41	14	27.292
Libya	LBY	46	47	36	48	8	19	13	47	27.704
Nigeria	NGA	14	24	28	27	31	41.5	40	28	27.889
Egypt	EGY	38	42	46	49	2	48	28	41	28.169
Ghana	GHA	37	19	25	23	40	22	44	29	28.650
South Africa	ZAF	45	40	49	40	15	16	17	48	30.195
Algeria	DZA	41	39	44	44	13	23	22	45	31.279
Morocco	MAR	35	35	43	41	14	29	37	40	32.670

We also tested the sensitivity of the final structural equation model results to variation in the minimum number of environmental indices used to construct the composite environmental performance index. Including all 8 environmental indices (ecological footprint^48^, megafauna conservation index^49^, relative species threat; freshwater removals; recent proportional forest loss^50^, livestock per hectare of arable land, extent of permanent croplands, greenhouse-gas emissions in 2013) reduced the number of countries in the ranking from 48 (Table 2) to 41 (Table 3).

**Table 3:** Ranking results (mandating that all environmental indices be available to calculate the composite environmental index; *n* = 41 countries).

Country	ISO	EF	MCI	THR	FWR	FRL	LVS	CPL	EMI	ENV_gm_
Cent Afr Rep	CAF	18	5	1	2	25	44	11	6	7.754
Botswana	BWA	44	1	4	31	10	7	1	42	7.955
Congo	COG	20	20	9	1	27	2	14	31	9.790
Dem Rep Congo	COD	3	33	23	5	43	10	21	2	10.943
Zambia	ZMB	8	6	8	18	39	11	7	21	12.020
Chad	TCD	31	23	11	26	19	13	5	3	12.876
Burundi	BDI	2	22	22	21	24	43	45	1	13.240
Mozambique	MOZ	5	10	38	12	46	4	20	15	13.724
Angola	AGO	6	17	12	10	38	7	15	39	14.454
Mali	MLI	30	29	5	34	16	30	10	5	15.623
Zimbabwe	ZWE	17	4	6	39	29	17	16	36	16.102
Rwanda	RWA	7	7	21	16	22	47	42	7	16.309
Niger	NER	32	26	15	38	6	24.5	9	9	16.557
Somalia	SOM	24	44	40	43	11	20	6	4	17.690
Lesotho	LSO	34	32	16	11	4	32	12	37	17.972
Malawi	MWI	4	9	37	32	33	35.5	30	8	18.190
Mauritania	MRT	42	46	27	46	7	15	2	33	18.950
Togo	TGO	13	12	10	15	35	33	36	26	19.970
Liberia	LBR	16	41	33	3	48	14	31	20	20.139
Burkina Faso	BFA	21	18	7	30	26	45	19	16	20.246
Ethiopia	ETH	11	21	29	35	17	46	29	10	21.913
Kenya	KEN	12	8	41	36	20	38	26	23	22.444
Sierra Leone	SLE	23	25	30	7	44	21	33	18	22.525
Gambia	GMB	9	38	14	22	32	41.5	23	22	22.711
Swaziland	SWZ	39	27	3	42	28	26	25	35	23.220
Tanzania	TZA	27	3	48	28	42	31	34	19	23.454
Cameroon	CMR	15	16	39	9	30	40	38	24	23.474
Senegal	SEN	19	13	26	33	21	37	18	32	23.557
Côte d’Ivoire	CIV	22	14	32	19	49	9	46	27	23.789
Guinea	GIN	29	36	34	8	36	18	35	17	23.984
Uganda	UGA	26	11	31	17	37	39	43	12	24.058
Benin	BEN	25	15	13	14	47	35.5	39	30	24.580
Tunisia	TUN	40	37	42	45	1	28	47	43	25.115
Guinea Bissau	GNB	33	43	19	13	45	34	41	14	27.292
Libya	LBY	46	47	36	48	8	19	13	47	27.704
Nigeria	NGA	14	24	28	27	31	41.5	40	28	27.889
Egypt	EGY	38	42	46	49	2	48	28	41	28.169
Ghana	GHA	37	19	25	23	40	22	44	29	28.650
South Africa	ZAF	45	40	49	40	15	16	17	48	30.195
Algeria	DZA	41	39	44	44	13	23	22	45	31.279
Morocco	MAR	35	35	43	41	14	29	37	40	32.670

**ISO** = Alpha-3 country code; **EF** = ecological footprint^48^; **MCI** = megafauna conservation index^49^; **THR** = relative species threat (number of IUCN Red List species classified as *Critically Endangered*, *Endangered*, *Vulnerable*, or *Near Threatened* divided by total number of species assessed; iucnredlist.org); **FWR** = freshwater removals (percent of internal resources; data.worldbank.org); **FRL** = recent (2000 to 2012) proportional forest loss^50^; **LVS** = livestock (cattle, pigs, buffaloes, sheep, and goats per hectare of arable land; fao.org/faostat); **CPL** = extent of permanent croplands (percent of total land area; data.worldbank.org); **EMI** = greenhouse-gas emissions (CO_2_-e per capita in 2013; data.worldbank.org).

### Structural equation models

**Figure 2:**
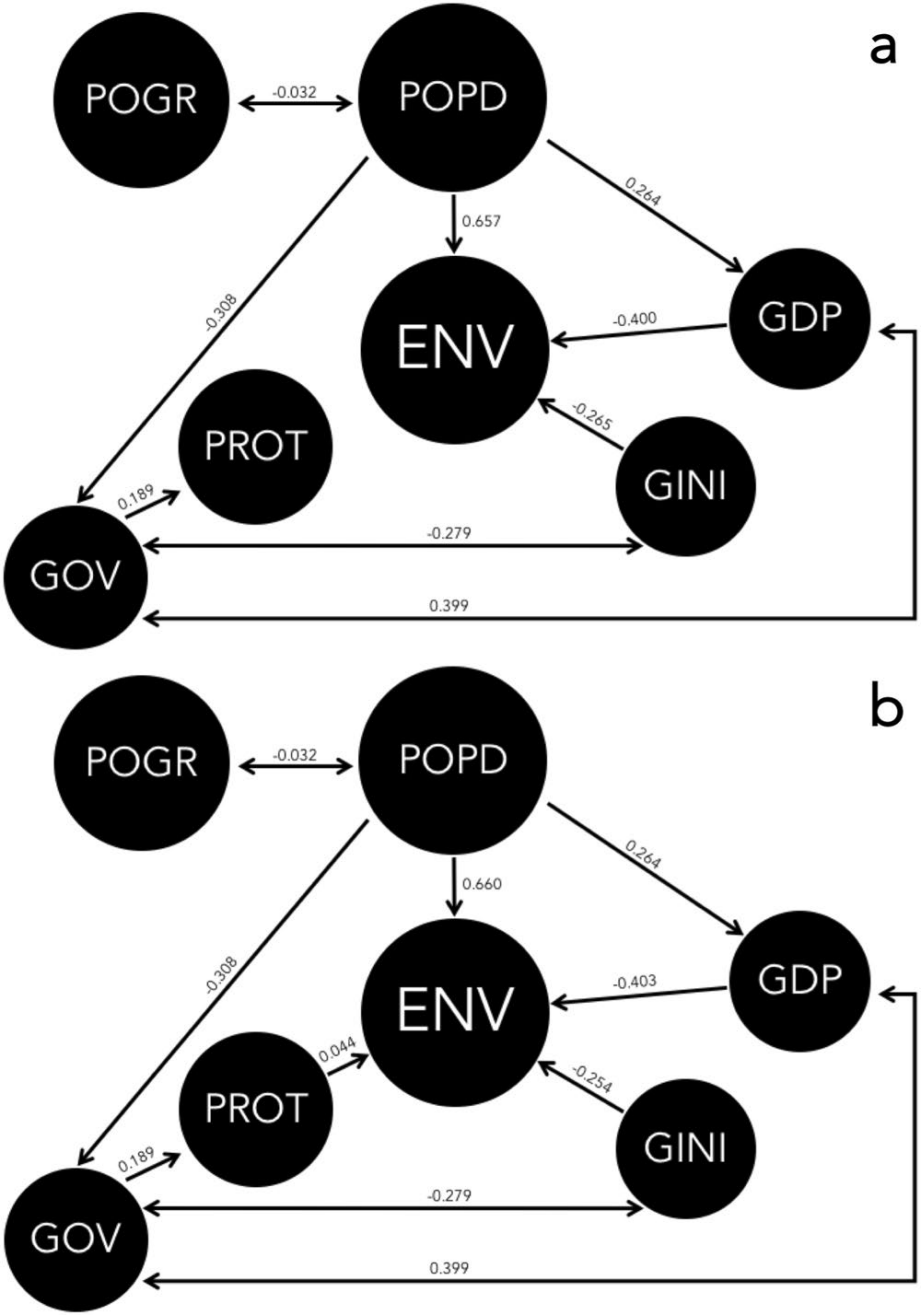
(**a**) Top-ranked structural equation model (in Table 3) where a nation’s environmental performance rank (**ENV**; low rank = best relative environmental performance) is positively correlated with population density (**POPD**), and negatively correlated with gross domestic product (**GDP**, corrected for purchasing-power parity), and Gini wealth inequality index (**GINI**). Numbers on the directional pathways indicate standardized coefficients for each relationship. (**b**) There is also some modest evidence for a positive effect of proportion of land area under protection (**PROT**) (see third-ranked model in Table 3). One-way and two-way correlations among predictor variables also shown. **POGR** = population growth rate.

**Figure 3:**
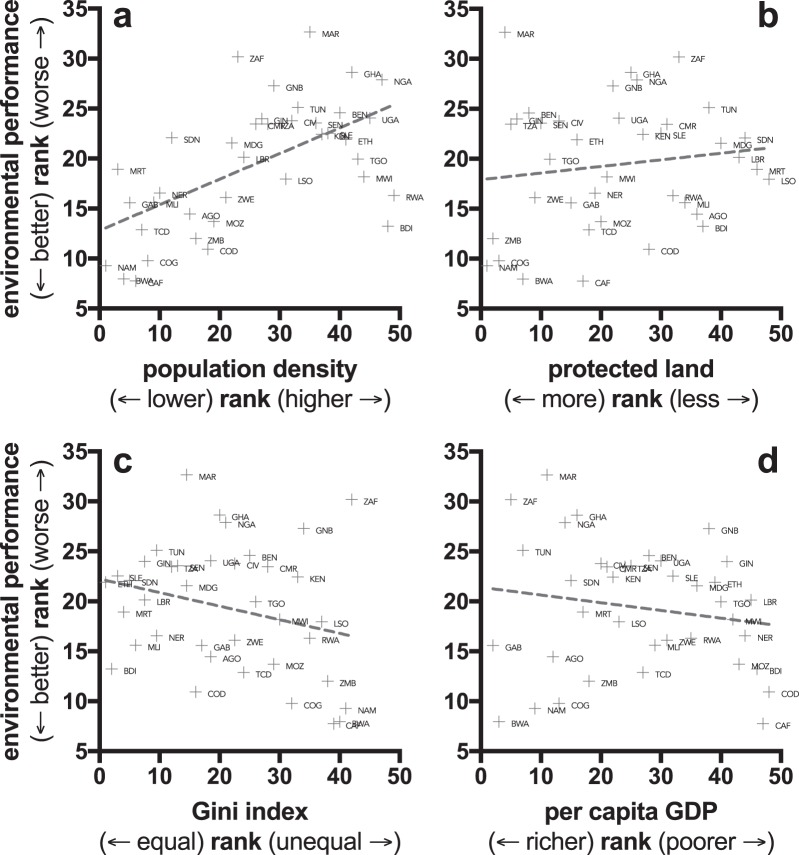
Bivariate rank relationships between (**a**) population density, (**b**) proportion of land area under protection, (**c**) Gini wealth distribution index, and (**d**) per capita GDP and relative environmental performance rank among African nations. Three-letter ISO country codes (point labels) are given in Table 2.

According to the thirteen structural equation models (Table 3; Fig. 2), the strongest predictor (i.e., appearing the most often in highest-ranked and highest goodness-of-fit models) of the composite environmental rank among African countries was population density (Table 3; see also Supplementary Information Methods and Results Section 3, Fig. S1 and Tables S3–S5, and Section 11, Fig. S5 for results from general linear mixed-effects models and boosted regression trees, respectively; these alternative modelling approaches takes either potential spatial autocorrelation or continuous responses into account, respectively), such that environmental performance (smaller rank) increased as a country’s population density decreased (Fig. 3a). While the top-ranked models with sufficient goodness-of-fit indicated that the land area under protection, wealth (GDP), and wealth disparity explained some additional variation in environmental rank (Table 4), the single-parameter explanatory models for these variables indicated weak relationships (Table 4; see also Supplementary Information Methods and Results Section 3, Tables S3–S5). Nonetheless, environmental rank improved to some extent as the proportion of the land area under protection increased (Fig. 3b), and it decreased as wealth distribution become more even (Fig. 3c) and per-capita GDP (wealth) increased. Re-running the structural equation models using the original configuration of the environmental performance index, but requiring all eight environmental variables in the calculation of the environmental performance rank (from Table 3), there was a slight shift in the top-ranked model (Table 5), but overall the main conclusions were still supported. This analysis resulted in 34 countries (cf. 38 countries for the less-stringent criterion of 7 of 8) environmental variables being considered (Table 5).

**Table 4:** Structural equation models considered in the model set correlating socio-economic variables to the composite geometric mean environmental ranking among countries (*n* = 38).

model	df	*χ* ^2^	ΔBIC	*w*BIC	NCI	IFI
POPD + GDP + GINI	**12**	**8.094**	**—**	**0.486**	**1.053**	**1.089**
POPD + GDP	**13**	**12.903**	**1.171**	**0.271**	**1.001**	**1.002**
POPD + GDP + GINI + PROT	**11**	**7.956**	**3.500**	**0.085**	**1.041**	**1.068**
POPD + GDP + GINI + GOV	**11**	**8.094**	3.637	**0.079**	**1.039**	**1.065**
POPD	14	19.775	4.405	0.054	0.927	0.862
POPD + PROT	13	19.333	7.601	0.011	0.920	0.853
POPD + GOV	13	19.350	7.618	0.011	0.920	0.852
*ALL*	**9**	**7.070**	**9.889**	**0.003**	**1.026**	**1.041**
GINI	14	32.840	17.470	<0.001	0.780	0.551
GOV	14	33.334	17.965	<0.001	0.775	0.540
GDP	14	34.497	19.128	<0.001	0.764	0.512
PROT	14	34.782	19.412	<0.001	0.761	0.505
POPG	14	34.807	19.437	<0.001	0.761	0.505

See Fig. 2 for a schematic of variable paths for the *All* model (including all variables). **POPD** = human population density; **GDP** = per capita gross domestic product (corrected for purchasing power parity); **GINI** = Gini wealth distribution index; **PROT** = proportion of land under some protection; **ALL** = model including all predictor variables; **GOV** = governance quality; **POPG** = human population growth rate. Values in the table refer to: **df** = degrees of freedom; ***χ***^**2**^ = chi-square; **ΔBIC** = difference in Bayesian information criterion of the top-ranked model and the model in question; ***w*BIC** = BIC model weight; **NCI** = McDonald’s non-centrality index (goodness-of-fit); **IFI** = Bollen’s incremental fit index (goodness-of-fit). All models with high goodness-of-fit (NCI and IFI > 0.9) in boldface.

**Table 5:** Structural equation models considered in the model set correlating socio-economic variables to the composite geometric mean environmental ranking among countries (*n* = 34; reduced set of countries from Table 3).

model	df	*χ* ^2^	ΔBIC	*w*BIC	NCI	IFI
POPD + GDP	**13**	**12.595**	—	**0.432**	**1.006**	**1.011**
POPD + GDP + GINI	**12**	**9.526**	**0.458**	**0.344**	**1.037**	**1.067**
POPD	14	20.029	3.908	0.061	0.915	0.829
POPD + GDP + GINI + GOV	**11**	**9.452**	**3.910**	**0.061**	**1.023**	**1.041**
POPD + GDP + GINI + PROT	**11**	**9.487**	**3.944**	**0.060**	**1.023**	**1.040**
POPD + GOV	13	18.379	5.784	0.024	0.924	0.851
POPD + PROT	13	20.027	7.432	0.011	0.902	0.806
*ALL*	**9**	**7.089**	**8.599**	**0.006**	**1.029**	**1.048**
GOV	14	28.801	12.680	0.001	0.804	0.579
GDP	14	30.577	14.456	<0.001	0.784	0.529
GINI	14	31.982	15.861	<0.001	0.768	0.489
POPG	14	32.028	15.907	<0.001	0.767	0.487
PROT	14	33.271	17.150	<0.001	0.753	0.452

**POPD** = human population density; **GDP** = per capita gross domestic product (corrected for purchasing power parity); **GINI** = Gini wealth distribution index; **PROT** = proportion of land under some protection; ***ALL*** = model including all predictor variables; **GOV** = governance quality; **POPG** = human population growth rate. Values in the table refer to: **df** = degrees of freedom; ***χ***^**2**^ = chi-square; **ΔBIC** = difference in Bayesian information criterion of the top-ranked model and the model in question; ***w*BIC** = BIC model weight^83^; **NCI** = McDonald’s non-centrality index^80^ (goodness-of-fit); **IFI** = Bollen’s incremental fit index^81^ (goodness-of-fit). All models with NCI and IFI > 0.9 in boldface.

## Discussion

It is simultaneously telling and disconcerting that none of the Sustainable Development Goal targets, nor any of the Aichi Biodiversity Targets, mentions reducing human population size as a pathway to achieving their goals, even though the United Nations promotes family planning as a means to empower people and develop nations^51^. Our finding that the strongest predictor of environmental performance among nations in Africa is population density means that countries with the most people per unit area suffered relatively more environmental degradation on average. This result brings into question the reality of the United Nations’ Sustainability Development Goals (www.un.org/sustainabledevelopment) — particularly Goal 15 (‘Sustainably manage forests, combat desertification, halt and reverse land degradation, halt biodiversity loss’), as well as the Convention on Biological Diversity’s Aichi Biodiversity Targets (www.cbd.int/sp/targets) Strategic Goals A (‘Address the underlying causes of biodiversity loss by mainstreaming biodiversity across government and society’) and B (‘Reduce the direct pressures on biodiversity and promote sustainable use’) — without dedicated, well-funded, and large-scale family planning rolled out across the African continent. Indeed, the targets for human development are becoming increasingly connected with those for natural systems and biodiversity^52^, and so we concur that the “… next generation of [human development and policy] scenarios should explore alternative pathways to reach these intertwined targets, including potential synergies and trade-offs between nature conservation and other development goals”^52^.

Combined with the stagnation of natural fertility decline in Africa compared to other developing regions of the world^10^, there has therefore never before been a more important time to re-invigorate the need for long-term, culturally sensitive, and meaningful family-planning measures if many African nations are to have any hope of stemming the decline of their biodiversity. This is particularly urgent for countries such as Nigeria (~187 million inhabitants in 2016; fertility = 5.5/woman; exponential rate of increase 2000–2015 = *r*_2000−15_ = 0.39), Democratic Republic of Congo (~80 million; fertility = 6.5/woman; *r*_2000−15_ = 0.48), South Africa (~56 million; fertility = 2.4/woman; *r*_2000−15_ = 0.22), Tanzania (~54 million; fertility = 5.2/woman; *r*_2000−15_ = 0.45), Kenya (~45 million; fertility = 3.9/woman; *r*_2000−15_ = 0.39), and Ghana (~28 million; fertility = 4.2/woman; *r*_2000−15_ = 0.38) (see also Fig. 1).

Fertility rates particularly in sub-Saharan Africa remain high, in part due to high poverty, low education^53^, and high child mortality^10^, thus resulting in a desire for large family sizes^54^. In Western Africa in particular, the adoption of contraception has been slow due to pervasive attitudinal resistance^55^, even though there is still considerable unmet demand^56,57^. As such, many national governments in Africa have not prioritised family-planning programs^54^; yet, well-designed family planning with regionally and culturally specific approaches (e.g., traditional methods, spacing designs)^56^ allows people to regulate their reproduction, with well-established benefits for family welfare^58^, national economies^58^, and the environment^54^. For example, countries like Botswana, South Africa, and Zimbabwe benefited from early adoption of population policies and family-planning programs^56^. One culprit for slow or stalled implementation elsewhere is that early deaths from the HIV/AIDS epidemic — while having limited demographic impact partly because of antiretroviral availability — have nonetheless shifted emphasis away from family planning^54^. It is therefore undeniable that African citizens and their governments would benefit from placing greater emphasis on quality family planning, a conclusion that we have also reached with respect to Africa’s environmental integrity^54^.

Some past investigations of the relationships between human population size/density and measures of environmental status have been equivocal^22–32,37^ suggesting that issues of spatial and temporal scale, as well as the choice of environmental indicator, have bearing on the strength of evidence arising. At the national scale in Africa, human population density most likely reflects the current state of environmental performance because of the relative uniformity among the sample of nations regarding the timing of principal environmental change, as well as the rapid recent expansion of human populations in many countries in that region^9,10^. A fundamental tenet of population ecology is that per-capita resources decline as populations near carrying capacity^59^, so the absolute pressure on the environment is dictated more by variation in a country’s ‘carrying capacity’ than absolute population size or per capita resource use *per se*^37^. Nonetheless, population density in the African context appears to be a reasonable reflection on average of an individual country’s proximity to this moving carrying-capacity target, despite localized improvements in biodiversity following fence construction^60^, for example.

Previous country rankings for environmental performance^37^ have not incorporated indices of *leakage* (externalizing environmental damage via pollution trading and outsourcing environmentally intensive production processes), although it is debatable whether it would make a large difference in the African context because of the relatively lower developed state of many of its nations compared to large consumers such as China, USA, and Brazil^37^. However, because we included each nation’s ecological footprint in our derivation of a composite environmental performance indicator, this should at least partially account for some aspects of leakage. Another potential caveat is that our modern ‘snapshot’ of the trends driving environmental degradation among African nations is likely to vary temporally, such that older comparisons could reveal alternate patterns. However, data for the variables we used to construct our analyses are largely unavailable and/or incomparable for periods vastly older than our current dataset.

It is unsurprising that per-capita wealth (GDP) had the hypothesised effect on a country’s relative environmental performance rank, especially considering that at the global scale at least, rising GDP reduces environmental performance among nations^37^. That same analysis^37^ also found no evidence to support the environmental Kuznets curve^61^ — the hypothesis that a U-shaped relationship exists between environmental degradation and per-capita wealth. This hypothesis predicts that beyond a certain threshold, wealthier societies begin to reduce their environmental footprints. However, the evidence for the environmental Kuznets curve is equivocal^62^, depending on which metrics are measured, countries examined, and periods of development history^61,63–70^. Examining the bivariate plot between environmental performance rank and per-capita GDP rank (Fig. 3d) might suggest a U-shaped relationship; however, examined appropriately by partialling the effects of the other socio-economic variables using a boosted regression tree approach that can identify nonlinearities, there is no evidence of a U-shaped relationship (Supplementary Information Methods and Results Section 12, Fig. S6).

It is not clear why governance quality consistently emerges as a weak predictor of environmental performance^37^. This conclusion exists even after using an African-specific indicator of governance quality^71^, possibly because governance problems in environmental custodianship might only become clear at finer spatial scales, perhaps only at regional or protected-area levels^32^. Alternatively, because governance quality tends to be ubiquitously low across the African continent relative to elsewhere^72^, the low inter-country variation in this metric likely diminishes the power to identify a correlation with environmental performance. The weak, yet statistically supported relationship between environmental rank and wealth disparity was as predicted — increasing wealth disparity leads to better environmental performance. This relationship might seem counter-intuitive, but there is evidence that when democratic processes are restricted, a less equal income distribution generates less environmental degradation^73,74^. The observed relationship most likely arises because greater inequality in wealth among citizens likely engenders fewer opportunities for development of natural resources, thus hindering or at least delaying environmental damage^45^.

In conclusion, our results strongly support the idea that a sustainable approach to biodiversity conservation in Africa over the coming decades cannot be limited by a narrow perspective that treats different development goals of well-being and environmental custodianship as separate entities if they ignore issues of sustained human population growth^52^. Indeed, with the mounting pressures facing Africa’s ecological systems, continued environmental degradation will impose further negative feedbacks on human well-being, because human quality of life is fundamentally tied to the healthy functioning of ecosystems^52^. Of course, better education, poverty alleviation, technological advances, and participation in multilateral environmental agreements could restrict land-use change and consumption rates and patterns; however, while there are many policy levers that African nations can use to improve the future state of their environments and the societies that depend on them, limiting excessive human population growth will, on average, likely facilitate better environmental custodianship.

## Methods

### Environmental data

Our goal was to define an African-relevant composite environmental indicator rank for each nation on the continent. While there are many ways to measure a nation’s environmental performance, there are more regionally and temporally relevant measures that attest to the specific environmental histories of regions. We therefore reasoned that given the recent colonial history of many African nations, the recent spike in human population sizes, rapid development investment over the last few decades, a rich diversity of megafauna under substantial threat from agricultural expansion and poaching^39^, and an emphasis on primary production (cropping, livestock husbandry), that the following available indicators would be ideal to construct a composite environmental index for African nations: *ecological footprint* (footprintnetwork.org), *megafauna conservation index*^49^, *IUCN Red List species threat* (iucnredlist.org), *freshwater removal* (data.worldbank.org), *forest loss*^50,75^, *livestock density* (fao.org/faostat), *cropland extent* (data.worldbank.org), and *greenhouse-gas emissions* (data.worldbank.org). We provide a full description of each indicator in the Supplementary Information (Section 1).

### Combined environmental performance indicator

For each environmental variable, we made simple hierarchical rankings (i.e., we did not consider the magnitude of the differences among absolute values between countries to avoid issues related to heteroscedasticity, non-linearity, and non-Gaussian distributions) using the *rank* function (means averaged) in R^76^. To construct a mean rank across all seven variables, we calculated geometric mean rankings for countries^37^ where at least seven of the eight variables were available to provide a measure of relative distance between countries in the final composite rank. We argue that a ‘seven out of eight’ criterion maximizes sample size (number of countries) without compromising the meaningfulness of the combined index (see Tables 3 and 5 for a sensitivity analysis of this choice). This ranking approach also avoids the undue influence of outliers (i.e., analogous to a geometric mean)^77^:

$${\rm{geometric}}\,{\rm{mean}}\,{\rm{rank}}={10}^{\sum _{i=1}^{k}\frac{{\mathrm{log}}_{10}({\rm{rank}}({x}_{i}))}{k}}$$

where *x*_*i*_ = environmental metric *i* (for *k* metrics considered).

### Socio-economic data

For a detailed description of the socio-economic variables and associated hypotheses, see Supplementary Information Section 2. In summary, we accessed the World Bank database for the estimated human population size for African nations in 2015, dividing this value by total land area per country to calculate a human population density (data.worldbank.org). We hypothesized that increasing human density would lead to greater pressure on environmental resources^25^, thus lowering a country’s environmental performance rank. We also calculated the mean annual human population growth rate from 1960 to 2015 for African nations from the World Bank (data.worldbank.org), hypothesizing that faster mean population growth would hasten the exploitation of a country’s resources relative to slower-growing nations^25^.

Also from the World Bank, we accessed each country’s gross domestic product (GDP) per capita (corrected for purchasing-power parity) as an index of total wealth. Some countries were missing GDP estimates for certain years, so we took the mean of values from 2011–2015 as an indication of mean per-capita GDP to maximize the sample size of countries considered. Previously, we showed that a country’s total wealth leads to a lower environmental performance (i.e., more degradation)^37^. Also from the World Bank, we accessed an index of wealth distribution using the Gini index from 2005 to 2014 (again, taking the mean of values across this period to maximize sample size). We hypothesized that the greater a country’s inequality in wealth across its citizenry, the lower the environmental damage that would ensue due to higher poverty and less overall development^45^.

We also hypothesized that poorer overall governance would lead to higher likelihood of environmental exploitation based on previous work linking it to environmental degradation^46,47^ (although at a global scale, declining governance quality had little impact on national-scale environmental performance)^37^. We used the Overall Governance Score from the 2015 Ibrahim Index of African Governance^71^, which includes measures of safety and rule of law, participation and human rights, sustainable economic opportunity, and human development indicators in its normalized overall score.

Finally, we hypothesized that a country’s commitment to protecting its native species, expressed through the proportion of its total land area under some form of protection, would lead to great environmental performance^33^. However, it is not part of the composite environmental performance indicator because the amount or number of protected areas does not necessarily translate into lower extinction rates^33^. To this end, we accessed the percentage of land under protected-area status for each country from the Population Reference Bureau (pbr.org), which is originally sourced from the World Database of Protected Areas (protectedplanet.net).

### Structural equation models

To account for inter-correlations among hypothesized socio-economic explanatory variables^37^, we applied structural (path) equation models to model the hypothesized relationships^78^. We constructed thirteen candidate models (see Results Table 3) to examine the socio-economic drivers of environmental rank among African countries, keeping the hypothesized relationships between socio-economic variables constant in all. These were: (a) a two-way correlation between human population density and growth rate, based on the assumption that compensatory density feedbacks operated between these two population variables; (b) a two-way correlation between governance score and per-capita GDP; (c) a two-way correlation between per-capita GDP and wealth distribution; (d) a one-way correlation between population density and per capita GDP; and (e) a one-way correlation between governance quality and the proportion of the landscape under some form of protection (see Results for schematic). Prior to fitting, we investigated the non-parametric ordinal rank correlations using Kendall’s *τ* because we used ranks in all models. We fitted the candidate path models to the data using the sem function^79^ implemented in the R Package^76^, calculating Bayesian information criterion (BIC) weights to assign relative strength of evidence to each model in the set. We evaluated the goodness-of-fit of each model using McDonald’s non-centrality index^80^ and Bollen’s incremental fit index^81^ using the semGOF library in R, both of which should be >0.90 to consider a model’s fit to be acceptable^81^. We also considered structural equation models using single environmental indicators to examine which elements of environmental change were most influenced by variation in socio-economic conditions (Supplementary Information Methods and Results Section 8, Table S10). We also considered only the ‘biodiversity’ components (i.e., *megafauna conservation index*^49^, *IUCN Red List species threat*, and *forest loss*^50,75^) to create a second composite environmental rank to determine its relationship to the socio-economic correlates in isolation from the other ‘agricultural’ (*freshwater removal*, *livestock density*, and *cropland extent*) and economic (*ecological footprint*, and *greenhouse-gas emissions*) components of the environmental performance rank (Supplementary Information Methods and Results Section 9, Table S11). We also considered a country’s poverty gap (percentage of people below the relevant country’s poverty threshold — data from the World Bank) instead of the Gini index as a measure of wealth inequality (Supplementary Information Methods and Results Section 10, Table S12). These models included fewer countries (34), had generally poorer fits, but supported the dominance of population density as the most important correlate (Table S12).

### Boosted regression trees

Finally, we considered the absolute differences between the values comprising the environmental performance metric, as well as those between the predictor values (*cf*. ranks) to examine whether ranking — despite its advantages for avoiding unequal variances, non-linearities, and non-Gaussian behaviour — resulted in substantially different conclusions. We therefore used the same data that we obtained to derive the rankings, but instead scaled and centred the data for each composite environmental metric, and then took the median value to derive a new, continuous-variable environmental-performance metric. Next, we scaled and centred the socio-economic predictor variables in the same manner, and then tested for relationships as we did for the ranked data. However, even scaling and centring could not remove potential problems of non-Gaussian distributions (Supplementary Information Methods and Results Section 11, Figs S2–S4), so we employed boosted regression trees^82^ instead to test the relationships (Supplementary Information Methods and Results Section 11, Fig. S5).

## Supplementary information

Supplementary Information Methods and Results
