# Supplementary material for: Socio-economic predictors of environmental performance among African nations
Source: Sci Rep. 2019 Jun 26;9:9306. doi: 10.1038/s41598-019-45762-3 (PMC6594960; doi:10.1038/s41598-019-45762-3)
Supplement: Supplementary file 1 — Supplementary Information Methods and Results [file 41598_2019_45762_MOESM1_ESM.pdf]

Socio-economic predictors of environmental performance among African nations

Corey J. A. Bradshaw & Enrico Di Minin

Supplementary Information Methods and Results

Table of contents

- **Section 1**: Description of environmental indicators to construct composite environmental performance index
- **Section 2**: Description of socio-economic variables and associated hypotheses
- **Section 3**: General linear mixed-effects models
- **Section 4**: Excluding ecological footprint
- **Section 5**: Considering Near Threatened species as non-threatened
- **Section 6**: Replacing forest loss with the forest harvest index
- **Section 7**: Considering croplands as a proportion of total arable area
- **Section 8**: Structural equation models with single environmental indices
- **Section 9**: Structural equation models considering a composite environmental rank composed only of the 'biodiversity' components
- **Section 10**: Structural equation models considering poverty gap instead of the Gini index as a measure of wealth inequality
- **Section 11**: Boosted regression trees testing hypotheses using continuous variables
- **Section 12**: Boosted regression tree fit for the relationship between environmental performance rank and per-capita GDP rank

Section 1: Description of environmental indicators to construct composite environmental performance index

(*i*) **Ecological footprint**. The ecological footprint (footprintnetwork.org) measures the ecological 'assets' that a particular human population requires to produce the natural resources it consumes and to absorb its wastes. Based on six categories (cropland, grazing land, fishing areas, built-up land, forest area, carbon demand on land), the *footprint* tracks the productive surface areas, and at the national scale, measures the relative ecological resource use per capita of a country via both domestic and foreign (via trade) consumption. While the use of the ecological footprint for ecological and policy implications might have limitations, such as its inability to represent the complexity of sustainable development[1], it appears to be a reasonable metric for assessing the relative global impact of individual countries. Nonetheless, we tested whether its exclusion from the composite environmental performance index would substantially alter our conclusions; its exclusion made little difference (Supplementary Information Section 4).

(*ii*) **Megafauna conservation index**. Africa is home to the world's last remaining megafauna (carnivores weighing ≥ 15 kg and herbivores ≥ 100 kg), and as such has a unique set of opportunities and challenges in biodiversity conservation. The recently devised megafauna conservation index[2] combines the ecological, protected area, and financial contributions toward megafauna conservation across countries standardised to a 0-100 scale. The megafauna conservation index essentially measures a country's performance and potential for preserving its megafauna diversity.

(*iii*) **Species threat**. The IUCN Red List (iucnredlist.org) is the international standard for assessing the relative threat risk of a region's biota. Summarised by country, we took for each country in Africa the total number of species (including all assessed mammals, birds, reptiles, amphibians, fishes, molluscs, other invertebrates, plants, fungi, and protists) assessed as 'threatened' (*Critically Endangered* + *Endangered* + *Vulnerable*) and Near Threatened as a proportion of the total number of species assessed in the Red List (i.e., excluding *Extinct*, *Extinct in the Wild*, *Least Concern* and *Data Deficient*) species (iucnredlist.org) from 2016. We also tested whether including Near Threatened species as *non-threatened* substantially altered our conclusions; it had little effect (Supplementary Information Section 5).

(*iv*) **Freshwater removal**. The world's freshwater biota is one of the most threatened groups on Earth because of rising water extraction, pollution, and wetland and waterway degradation[3,4]. As a gross index of relative freshwater removal for each African country, we accessed data from the World Bank (data.worldbank.org) on the 2014 annual freshwater withdrawals (including water from desalination plants, but not counting evaporation losses from storage basins; if 2014 values were absent, we used either the 2002 or 2007 values, whichever was most recent). Withdrawals also include agricultural and industrial uses (including irrigation, water for livestock production), water for industrial uses, drinking

water, municipal use/supply, and water for public services, establishments and homes, and are expressed as a percentage of available internal freshwater resources.

(*v*) **Forest loss**. Loss of forest cover from agricultural expansion, timber harvest, and urbanisation is one of the principal threatening processes of biodiversity extinction[5], and in Africa in particular, large-scale deforestation is a relatively modern phenomenon arising from foreign investment, rapid population growth, and large-scale agricultural endeavours[6]. We therefore accessed the most up-to-date estimates of recent (2000-2012) forest-loss data from Hansen et al.[7], expressing the change in forest cover as proportions of forest area in 2000. We also used a second metric of forest use — the forest harvest index[8] (data compiled for 2000-2004) — to examine whether this more direct measure of forest exploitation (in terms of industrial roundwood and wood fuel harvest) altered our conclusions; using the forest harvest index instead had little effect on our conclusions (Supplementary Information Section 6).

(*vi*) **Livestock density**. The entire African continent is estimated to have around 260 million head of livestock (from FAO livestock data for 2011 and World Bank arable-land data for 2014)[9], which represent a major component of environment degradation[10]. Using data from the Food and Agriculture Organization of the United Nations (fao.org), we calculated the number of cattle, pigs, buffaloes, sheep, and goats per hectare of arable land per country for the most recent year available (2011; fao.org/faostat).

(*vii*) **Cropland extent**. Agricultural expansion, along with livestock grazing pressure, is one of the major threatening processes to biodiversity worldwide[5]. We therefore accessed data on the proportion of each African nation's total land area dedicated to permanent cropland (2014 data; data.worldbank.org). We also repeated our analyses using cropland extent as a proportion of total arable land area per country (with little effect to our conclusions; Supplementary Information Section 7).

(*viii*) **Greenhouse-gas emissions**. Although the Ecological Footprint includes a carbon footprint, it expresses this as the area of forest land required to sequester anthropogenic carbon dioxide emissions. This, combined with the low correlation between Ecological Footprint and total carbon-dioxide equivalents (CO_2-e) emissions data for countries (see Table 1), argues for including this metric in the environmental performance assessment. Thus, we accessed data from the World Bank quantifying the per capita CO_2-e emissions per country in 2013 (data.worldbank.org).

Section 2: Description of socio-economic variables and associated hypotheses

We accessed the World Bank database for the estimated human population size for African nations in 2015, dividing this value by total land area per country to calculate a **human population density** (data.worldbank.org). We hypothesised that increasing human density would lead to greater pressure on environmental resources[11], thus lowering a country's environmental performance rank. We also calculated the mean annual human population growth rate from 1960 to 2015 for African nations from the World Bank (data.worldbank.org), hypothesising that faster mean population growth would hasten the exploitation of a country's resources relative to slower-growing nations[11].

Also from the World Bank, we accessed each country's **gross domestic product** (GDP) per capita (corrected for purchasing-power parity) as an index of total wealth. Some countries were missing GDP estimates for certain years, so we took the mean of values from 2011-2015 as an indication of mean per-capita GDP to maximise the sample size of countries considered. Previously, we showed that the greater a country's total wealth, the lower its environmental performance (i.e., more degradation)[12]. Also from the World Bank, we accessed an index of wealth distribution using the **Gini index** from 2005 to 2014 (again, taking the mean of values across this period to maximise sample size). We hypothesised that the greater a country's inequality in wealth across its citizenry, the lower the environmental damage that would ensue due to higher poverty and less overall development[13].

We also hypothesised that poorer overall governance would lead to higher likelihood of environmental exploitation based on previous work linking it to environmental degradation[14,15] (although at a global scale, declining governance quality had little impact on national-scale environmental performance)[12]. We used the **Overall Governance Score** from the 2015 Ibrahim Index of African Governance[16], which includes measures of safety and rule of law, participation and human rights, sustainable economic opportunity, and human development indicators in its normalised overall score.

Finally, we hypothesised that the greater a country's commitment to protecting its native species, expressed through the **proportion of its total land area under some form of protection**, the higher would be its environmental performance[17]. However, it is not part of the composite environmental performance indicator because the amount or number of protected areas does not necessarily translate into lower extinction rates[17]. To this end, we accessed the percentage of land under protected-area status for each country from the Population Reference Bureau (pbr.org), which is originally sourced from the World Database of Protected Areas (protectedplanet.net).

Section 3: General linear mixed-effects models

In addition to the structural equation models, we employed general linear mixed-effects models (GLMM) with a Gaussian error distribution and identity links (log-link Gaussian models failed to converge). We applied the `lmer` function from the *lme4* package[18] in R, with a 'region' random effect according to three different regionalisations for the African continent to account for any broad-scale spatial non-independence. Although including all data in this way ignores other non-independence issues (e.g., country adjacencies), it identifies which correlates are likely to provide some explanatory power. The three regionalisation schemes we used were (*a*) the five United Nations regions (northern, western, middle, eastern, southern; www.un.org), (*b*) the five African Union regions (north, west, central, east, southern; www.au.int), and (*c*) the two World Health Organization regions (Africa D [~ northwest] and Africa E [~ southeast]; www.who.int) (Fig. S1).

We ranked 13 candidate models for each regionalisation random-effect scenario using Akaike's information criterion[19] (AIC), expressing model probability as an AIC weight (*w*AIC)[20]. We also calculated the marginal R^2 of each resampled GLMM (R_m) as a measure of goodness of fit and the contribution of the fixed effects to explaining variance in the response variable (environmental performance rank)[21]. We fit all models to the original configuration of the data presented in the main text. Model rankings and associated metrics are given in Tables S3-S5).

Figure S1. Three regionalisation schemes according to the (*a*) United Nations, (*b*) African Union, and (*c*) World Health Organization.

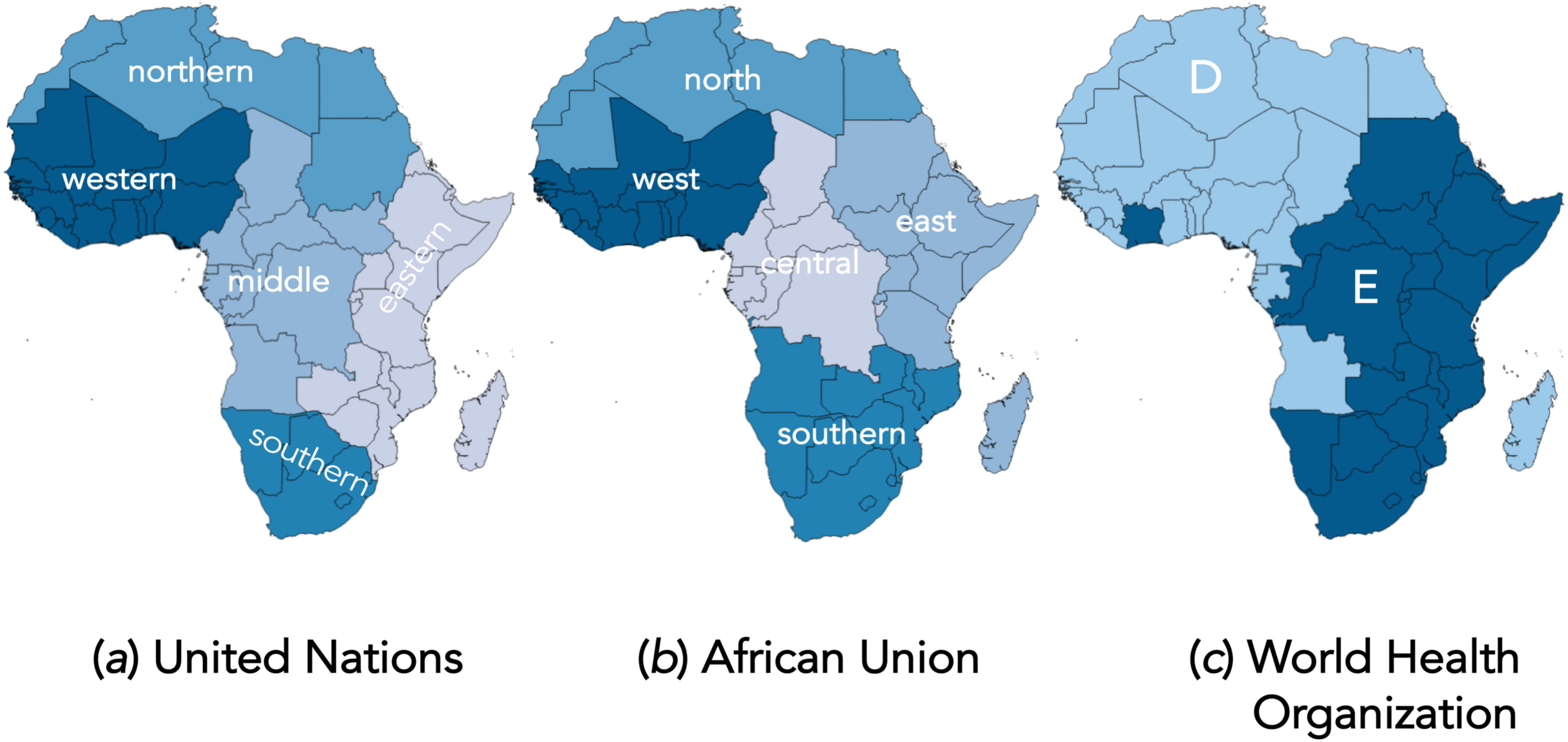

Table S3. General linear mixed-effects models considered in the model set correlating socio-economic variables to the composite geometric mean environmental ranking among countries (n = 38). All models include a random effect following the United Nations regions (northern, western, middle, eastern, southern; Fig. S1*a*). **POPD** = human population density; **GDP** = per capita gross domestic product (corrected for purchasing power parity); **GINI** = Gini wealth distribution index; **PROT** = proportion of land under some protection; ***ALL*** = model including all predictor variables; ***intercept-only*** = model including only the intercept (i.e., no fixed effects); **GOV** = governance quality. Values in the table refer to: ***k*** = number of model parameters; **LL** = log-likelihood, **ΔAIC** = difference in Akaike's information criterion[19] of the top-ranked model and the model in question; ***w*AIC** = AIC model weight[20]; $\mathbf{R_m}$ = marginal R^2 as a measure of goodness-of-fit[21].

model	*k*	LL	ΔAIC	*w*AIC	R_m
POPD+GDP+GINI	6	-114.013	-	0.328	43.931
POPD+GDP	5	-112.924	0.106	0.311	37.082
POPD+GDP+GINI+PROT	7	-115.886	1.784	0.134	43.283
POPD+GDP+GINI+GOV	7	-115.662	1.938	0.124	42.010
ALL	8	-117.532	3.749	0.050	41.520
POPD	4	-114.149	4.884	0.029	29.456
POPD+GOV	5	-115.781	6.545	0.012	29.815
POPD+PROT	5	-116.052	6.683	0.012	29.529
GDP	4	-119.979	17.559	<0.001	5.509
intercept-only	3	-119.607	18.169	<0.001	-
GOV	4	-120.507	18.620	<0.001	3.596
PROT	4	-121.182	19.644	<0.001	0.807
GINI	4	-121.096	19.922	<0.001	0.293

Table S4. General linear mixed-effects models considered in the model set correlating socio-economic variables to the composite geometric mean environmental ranking among countries (n = 38). All models include a random effect following the African Union regions (north, west, central, east, southern; Fig. S1*b*). **POPD** = human population density; **GDP** = per capita gross domestic product (corrected for purchasing power parity); **GINI** = Gini wealth distribution index; **PROT** = proportion of land under some protection; ***ALL*** = model including all predictor variables; ***intercept-only*** = model including only the intercept (i.e., no fixed effects); **GOV** = governance quality. Values in the table refer to: ***k*** = number of model parameters; **LL** = log-likelihood, **ΔAIC** = difference in Akaike's information criterion[19] of the top-ranked model and the model in question; ***w*****AIC** = AIC model weight[20]; $\mathbf{R_m}$ = marginal R^2 as a measure of goodness-of-fit[21].

model	*k*	LL	ΔAIC	*w*AIC	R_m
POPD+GDP	5	-112.283	-	0.411	31.466
POPD+GDP+GINI	6	-113.736	0.918	0.260	36.617
POPD+GDP+GINI+GOV	7	-115.169	2.385	0.125	33.108
POPD+GDP+GINI+PROT	7	-115.524	2.449	0.121	36.409
ALL	8	-117.023	4.035	0.055	33.376
POPD	4	-114.290	6.515	0.016	19.790
POPD+PROT	5	-116.152	8.233	0.007	20.341
POPD+GOV	5	-116.078	8.407	0.006	19.909
GDP	4	-118.218	15.101	<0.001	4.496
intercept-only	3	-117.736	15.864	<0.001	-
GOV	4	-118.971	16.726	<0.001	2.170
PROT	4	-119.412	17.450	<0.001	0.583
GINI	4	-119.323	17.863	<0.001	0.048

Table S5. General linear mixed-effects models considered in the model set correlating socio-economic variables to the composite geometric mean environmental ranking among countries (*n* = 38). All models include a random effect following the World Health Organization regions (Africa D, Africa E; Fig. S1*c*). **POPD** = human population density; **GDP** = per capita gross domestic product (corrected for purchasing power parity); **GINI** = Gini wealth distribution index; **PROT** = proportion of land under some protection; ***ALL*** = model including all predictor variables; ***intercept-only*** = model including only the intercept (i.e., no fixed effects); **GOV** = governance quality. Values in the table refer to: ***k*** = number of model parameters; **LL** = log-likelihood, **ΔAIC** = difference in Akaike's information criterion[19] of the top-ranked model and the model in question; ***w*AIC** = AIC model weight[20]; $\mathbf{R_m}$ = marginal R^2 as a measure of goodness-of-fit[21].

model	*k*	LL	ΔAIC	*w*AIC	R_m
POPD+GDP	5	-112.278	-	0.354	39.122
POPD+GDP+GINI	6	-113.460	0.224	0.316	43.202
POPD+GDP+GINI+PROT	7	-115.409	2.113	0.123	42.878
POPD+GDP+GINI+GOV	7	-115.268	2.189	0.118	42.567
ALL	8	-117.185	4.057	0.047	42.224
POPD	4	-113.999	5.802	0.019	30.017
POPD+GOV	5	-115.137	6.277	0.015	30.548
POPD+PROT	5	-115.892	7.675	0.008	29.947
GOV	4	-120.976	20.574	<0.001	7.608
intercept-only	3	-121.249	22.424	<0.001	-
GINI	4	-122.372	23.156	<0.001	2.301
GDP	4	-122.503	23.441	<0.001	1.928
PROT	4	-122.771	23.982	<0.001	0.782

Section 4: Excluding ecological footprint

We examined whether including the ecological footprint from the composite environmental performance rank affects our conclusions, due to the possibility of double-counting carbon emissions (despite the low correlation [Kendall's $\tau = 0.385$] between CO_2-e emissions and ecological footprint ranks among countries; Table 1). Re-running the structural equation models, Table S6 shows that conclusions are robust to the exclusion of Ecological Footprint, with the identical top-ranked model, and only small variation in the model ranks (cf. Table 3 and Table S6).

Table S6. Structural equation models considered in the model set correlating socio-economic variables to the composite geometric mean environmental ranking (excluding ecological footprint) among countries ($n = 36$). **POPD** = human population density; **GDP** = per capita gross domestic product (corrected for purchasing power parity); **GINI** = Gini wealth distribution index; **PROT** = proportion of land under some protection; ***ALL*** = model including all predictor variables; **GOV** = governance quality; **POPG** = human population growth rate. Values in the table refer to: **df** = degrees of freedom; χ^2 = chi-square; **ΔBIC** = difference in Bayesian information criterion of the top-ranked model and the model in question; ***w*BIC** = BIC model weight[20]; **NCI** = McDonald's non-centrality index[22] (goodness-of-fit); **IFI** = Bollen's incremental fit index[23] (goodness-of-fit). All models with NCI and IFI > 0.9 in boldface.

model	df	χ^2	ΔBIC	*w*BIC	NCI	IFI
**POPD+GDP**	**13**	**12.970**	**-**	**0.356**	**1.000**	**1.001**
**POPD+GDP+GINI**	**12**	**9.423**	**0.037**	**0.350**	**1.036**	**1.050**
**POPD**	**14**	**18.628**	**2.075**	**0.126**	**0.938**	**0.907**
**POPD+GDP+GINI+GOV**	**11**	**9.333**	**3.531**	**0.061**	**1.023**	**1.032**
**POPD+GDP+GINI+PROT**	**11**	**9.423**	**3.620**	**0.058**	**1.022**	**1.030**
POPD+GOV	13	18.272	5.303	0.025	0.929	0.896
POPD+PROT	13	18.599	5.629	0.021	0.925	0.890
ALL	**9**	**8.627**	**9.991**	**0.002**	**1.005**	**1.007**
GINI	14	43.199	26.645	<0.001	0.667	0.413
GOV	14	43.663	27.110	<0.001	0.662	0.404
PROT	14	45.094	28.541	<0.001	0.649	0.375
POPG	14	45.369	28.816	<0.001	0.647	0.369
GDP	14	45.560	29.007	<0.001	0.645	0.366

Section 5: Considering Near Threatened species as non-threatened

In the original analysis (main text), we considered the IUCN Red List (iucnredlist.org) category 'Near Threatened' as threatened to calculate relative species threat within the composite environmental index. In the following analysis, we re-calculated this proportion *excluding* Near Threatened species (i.e., number of IUCN Red List species classified as *Critically Endangered*, *Endangered*, or *Vulnerable* divided by total number of species assessed, including *Near Threatened*) and re-ran the structural equation models. The results are nearly identical to the original analysis (cf. Table S7 and Table 3).

Table S7. Structural equation models considered in the model set correlating socio-economic variables to the composite geometric mean environmental ranking (altered species threat calculation) among countries (n = 38). **POPD** = human population density; **GDP** = per capita gross domestic product (corrected for purchasing power parity); **GINI** = Gini wealth distribution index; **PROT** = proportion of land under some protection; ***ALL*** = model including all predictor variables; **GOV** = governance quality; **POPG** = human population growth rate. Values in the table refer to: **df** = degrees of freedom; χ^2 = chi-square; **ΔBIC** = difference in Bayesian information criterion of the top-ranked model and the model in question; ***w*BIC** = BIC model weight[20]; **NCI** = McDonald's non-centrality index[22] (goodness-of-fit); **IFI** = Bollen's incremental fit index[23] (goodness-of-fit). All models with NCI and IFI > 0.9 in boldface.

model	df	χ^2	ΔBIC	*w*BIC	NCI	IFI
**POPD+GDP+GINI**	**12**	**7.898**	**-**	**0.511**	**1.055**	**1.093**
**POPD+GDP**	**13**	**12.948**	**1.412**	**0.252**	**1.001**	**1.001**
**POPD+GDP+GINI+GOV**	**11**	**7.871**	**3.611**	**0.084**	**1.042**	**1.069**
**POPD+GDP+GINI+PROT**	**11**	**7.872**	**3.611**	**0.084**	**1.042**	**1.069**
POPD	14	19.908	4.735	0.048	0.925	0.860
POPD+GOV	13	19.608	8.072	0.009	0.917	0.847
POPD+PROT	13	19.655	8.119	0.009	0.916	0.846
ALL	**9**	**7.070**	**10.085**	**0.003**	**1.026**	**1.041**
GINI	14	33.002	17.828	<0.001	0.779	0.550
GOV	14	33.837	18.663	<0.001	0.770	0.531
GDP	14	34.756	19.583	<0.001	0.761	0.509
POPG	14	35.194	20.021	<0.001	0.757	0.499
PROT	14	35.259	20.086	<0.001	0.756	0.497

Section 6: Replacing forest loss with the forest harvest index

Our forest loss index by itself might not necessarily reflect the economic and social utility of forests for national economies and forest-dependent communities. We therefore replaced our index of forest loss[7] with the forest harvest index[8] that combines industrial roundwood and wood fuel data for 2000 to 2004 (ha year^{-1}). We summed the forest harvest index for both wood sources (roundwood and wood fuel), which is positively correlated with gross forest cover loss[7] (ha year^{-1}) for African nations as a power relationship (adjusted $R^2 = 0.32$; $\beta = 0.414 \pm 0.093$).

We re-ran the structural equation models with the updated environmental performance index using the combined forest harvest index, and the results (Table S8) were broadly consistent with the original results (i.e., same top-ranked model; minor ranking changes; cf. Table S8 and Table 3).

Table S8. Structural equation models considered in the model set correlating socio-economic variables to the composite geometric mean environmental ranking (replacing forest loss with the forest harvest index) among countries ($n = 35$). **POPD** = human population density; **GDP** = per capita gross domestic product (corrected for purchasing power parity); **GINI** = Gini wealth distribution index; **PROT** = proportion of land under some protection; ***ALL*** = model including all predictor variables; **GOV** = governance quality; **POPG** = human population growth rate. Values in the table refer to: **df** = degrees of freedom; χ^2 = chi-square; **ΔBIC** = difference in Bayesian information criterion of the top-ranked model and the model in question; ***w*BIC** = BIC model weight[20]; **NCI** = McDonald's non-centrality index[22] (goodness-of-fit); **IFI** = Bollen's incremental fit index[23] (goodness-of-fit). All models with NCI and IFI > 0.9 in boldface.

model	df	χ^2	ΔBIC	*w*BIC	NCI	IFI
**POPD+GDP+GINI**	**12**	**11.909**	**-**	**0.495**	**1.001**	**1.002**
POPD+GDP	13	17.493	2.028	0.180	0.938	0.885
**POPD+GDP+GINI+GOV**	**11**	**10.577**	**2.223**	**0.163**	**1.006**	**1.010**
**POPD+GDP+GINI+PROT**	**11**	**11.650**	**3.296**	**0.095**	**0.991**	**0.984**
ALL	**9**	**6.807**	**5.564**	**0.031**	**1.032**	**1.051**
POPD+GOV	13	22.529	7.065	0.014	0.873	0.756
POPD	14	26.302	7.282	0.013	0.839	0.677
GOV	14	28.636	9.617	0.004	0.811	0.616
POPD+PROT	13	25.881	10.417	0.003	0.832	0.670
GDP	14	31.076	12.056	0.001	0.784	0.551
POPG	14	33.272	14.253	<0.001	0.759	0.494
GINI	14	33.357	14.337	<0.001	0.758	0.491
PROT	14	35.021	16.001	<0.001	0.741	0.448

Section 7: Considering croplands as a proportion of total arable area

In the original analysis (main text), we considered croplands as a proportion of total land area to calculate relative species threat within the composite environmental index. In the following analysis, we re-calculated this proportion using the total amount of arable land per country to account partially for intensification. The results are nearly identical to the original analysis (cf. Table S9 and Table 3).

Table S9. Structural equation models considered in the model set correlating socio-economic variables to the composite geometric mean environmental ranking (altered croplands proportion) among countries (n = 38). **POPD** = human population density; **GDP** = per capita gross domestic product (corrected for purchasing power parity); **GINI** = Gini wealth distribution index; **PROT** = proportion of land under some protection; ***ALL*** = model including all predictor variables; **GOV** = governance quality; **POPG** = human population growth rate. Values in the table refer to: **df** = degrees of freedom; χ^2 = chi-square; **ΔBIC** = difference in Bayesian information criterion of the top-ranked model and the model in question; ***w*BIC** = BIC model weight[20]; **NCI** = McDonald's non-centrality index[22] (goodness-of-fit); **IFI** = Bollen's incremental fit index[23] (goodness-of-fit). All models with NCI and IFI > 0.9 in boldface.

model	df	χ^2	ΔBIC	*w*BIC	NCI	IFI
**POPD+GDP+GINI**	**12**	**8.647**	**-**	**0.522**	**1.045**	**1.081**
**POPD+GDP**	**13**	**13.929**	**1.644**	**0.229**	**0.988**	**0.977**
**POPD+GDP+GINI+PROT**	**11**	**8.125**	**3.116**	**0.110**	**1.039**	**1.068**
**POPD+GDP+GINI+GOV**	**11**	**8.620**	**3.611**	**0.086**	**1.032**	**1.056**
POPD	14	21.466	5.544	0.033	0.906	0.810
POPD+PROT	13	20.552	8.267	0.008	0.905	0.813
POPD+GOV	13	21.122	8.837	0.006	0.899	0.799
ALL	**9**	**7.070**	**9.336**	**0.005**	**1.026**	**1.043**
GINI	14	30.052	14.130	<0.001	0.810	0.592
GDP	14	31.077	15.155	<0.001	0.799	0.566
GOV	14	31.115	15.193	<0.001	0.798	0.565
PROT	14	31.673	15.751	<0.001	0.793	0.551
POPG	14	31.948	16.026	<0.001	0.790	0.544

Section 8: Structural equation models with single environmental indices

To examine which components of the composite environmental performance index were most likely responsible for the combined results (main text Table 3), we repeated the structural equation models using each component index separately. We scaled and centred the raw data (i.e., not ranks), and $\log_{10}$-transformed the ecological footprint, freshwater removals, cropland extent, and emissions, and logit-transformed the megafauna conservation index, to normalise the distributions because we did not use ranks in these cases.

The results for the two top-ranked structural equation models (or for more such that their combined Σ*w*BIC > 0.5) for each environmental index are shown in Table S10. Six of the 8 indices had a strong correlation to population density (ecological footprint, threatened species, forest loss, livestock, croplands, emissions), 5 of 8 to GDP (ecological footprint, freshwater removals, forest loss, livestock, emissions), and 1 to the Gini index (megafauna conservation index) (Table S10). Indeed, the overall pattern shows that individually, human population density and GDP were the most important correlates (cf. Table 3).

Table S10. Structural equation model results for single-variable environmental responses (ecological footprint, megafauna conservation index, threatened species, freshwater removals, forest loss, livestock, croplands, or emissions) relative to socio-economic variables. For each response, only the top-ranked models are shown (at least two top-ranked models, or those whose Σ*w*BIC > 0.5). **POPD** = human population density; **GDP** = per capita gross domestic product (corrected for purchasing power parity); **GINI** = Gini wealth distribution index; **PROT** = proportion of land under some protection; ***ALL*** = model including all predictor variables; **GOV** = governance quality; **POPG** = human population growth rate. Values in the table refer to: **df** = degrees of freedom; χ^2 = chi-square; **ΔBIC** = difference in Bayesian information criterion of the top-ranked model and the model in question; ***w*BIC** = BIC model weight[20]; **NCI** = McDonald's non-centrality index[22] (goodness-of-fit); **IFI** = Bollen's incremental fit index[23] (goodness-of-fit).

model	df	χ^2	ΔBIC	*w*BIC	NCI	IFI
ecological footprint						
GDP	14	21.707	-	0.381	0.901	0.820
POPD+GDP	13	18.181	0.085	0.365	0.932	0.882
megafauna conservation index						
PROT	14	20.065	-	0.576	0.921	0.839
GINI	14	22.727	2.662	0.152	0.889	0.769
threatened species						
PROT	14	11.306	-	0.407	1.035	1.133
POPG	14	13.358	2.052	0.146	1.008	1.032
freshwater removals						
GDP	14	17.344	-	0.297	0.958	0.873
GOV	14	18.368	1.024	0.178	0.946	0.834

	POPG	14	19.418	2.074	0.105	0.933	0.795
forest loss							
	GDP	14	11.589	-	0.395	1.031	1.111
	POPD	14	13.374	1.785	0.162	1.008	1.029
livestock							
	POPD	14	13.610	-	0.413	1.005	1.011
	POPD+GDP	13	10.780	0.782	0.280	1.030	1.060
croplands							
	POPD	14	14.624	-	0.442	0.992	0.990
	POPD+GOV	13	11.387	0.400	0.362	1.021	1.025
emissions							
	GDP	14	15.065	-	0.786	0.986	0.983
	POPD+GDP	13	14.611	3.210	0.158	0.980	0.975

Section 9: Structural equation models considering a composite environmental rank composed only of the 'biodiversity' components

Table S11. Structural equation models considered in the model set correlating socio-economic variables to the composite geometric mean environmental ranking composed only of the 'biodiversity' components (*megafauna conservation index*, *IUCN Red List species threat*, and *forest loss*) among countries (n = 38). **POPD** = human population density; **GDP** = per capita gross domestic product (corrected for purchasing power parity); **GINI** = Gini wealth distribution index; **PROT** = proportion of land under some protection; **ALL** = model including all predictor variables; **GOV** = governance quality; **POPG** = human population growth rate. Values in the table refer to: **df** = degrees of freedom; χ^2 = chi-square; **ΔBIC** = difference in Bayesian information criterion of the top-ranked model and the model in question; ***w*BIC** = BIC model weight; **NCI** = McDonald's non-centrality index (goodness-of-fit); **IFI** = Bollen's incremental fit index (goodness-of-fit). All models with high goodness-of-fit (NCI and IFI > 0.9) in boldface.

model	df	χ^2	ΔBIC	*w*BIC	NCI	IFI
**GINI**	**14**	**13.233**	**-**	**0.263**	**1.010**	**1.031**
**POPD+GOV**	**13**	**10.869**	**1.273**	**0.139**	**1.028**	**1.083**
**POPD**	**14**	**14.682**	**1.449**	**0.128**	**0.991**	**0.972**
**PROT**	**14**	**14.766**	**1.553**	**0.122**	**0.990**	**0.969**
**POPD+PROT**	**13**	**11.493**	**1.897**	**0.102**	**1.020**	**1.059**
**GDP**	**14**	**15.896**	**2.663**	**0.070**	**0.975**	**0.923**
GOV	14	16.649	3.415	0.048	0.966	0.893
**POPD+GDP+GINI**	**12**	**9.731**	**3.773**	**0.040**	**1.030**	**1.085**
**POPD+GDP**	**13**	**13.412**	**3.816**	**0.039**	**0.995**	**0.984**
POPG	14	18.214	4.981	0.022	0.946	0.830
**POPD+GDP+GINI+PROT**	**11**	**8.041**	**5.720**	**0.015**	**1.040**	**1.107**
**POPD+GDP+GINI+GOV**	**11**	**8.505**	**6.184**	**0.012**	**1.033**	**1.090**
ALL	**9**	**7.070**	**12.025**	**0.001**	**1.026**	**1.065**

Section 10: Structural equation models considering poverty gap instead of the Gini index as a measure of wealth inequality

Table S12. Structural equation models considered in the model set correlating socio-economic variables to the composite geometric mean environmental ranking countries (*n* = 38). **POPD** = human population density; **GDP** = per capita gross domestic product (corrected for purchasing power parity); **POVGAP** = poverty gap (percentage of people living below the relevant nation's poverty threshold — data from the World Bank); **PROT** = proportion of land under some protection; **ALL** = model including all predictor variables; **GOV** = governance quality; **POPG** = human population growth rate. Values in the table refer to: **df** = degrees of freedom; χ^2 = chi-square; **ΔBIC** = difference in Bayesian information criterion of the top-ranked model and the model in question; ***w*BIC** = BIC model weight; **NCI** = McDonald's non-centrality index (goodness-of-fit); **IFI** = Bollen's incremental fit index (goodness-of-fit). All models with high goodness-of-fit (NCI and IFI > 0.9) in boldface.

model	df	χ^2	ΔBIC	*w*BIC	NCI	IFI
**POPD+GDP+POVGAP**	**12**	**12.934**	**-**	**0.417**	**0.986**	**0.978**
**POPD+GDP**	**13**	**16.807**	**0.346**	**0.351**	**0.946**	**0.909**
**POPD+GDP+POVGAP+GOV**	**11**	**12.429**	**3.021**	**0.092**	**0.979**	**0.967**
**POPD+GDP+POVGAP+PROT**	**11**	**12.444**	**3.036**	**0.091**	**0.979**	**0.967**
POPD	14	25.274	5.287	0.030	0.847	0.723
POPD+GOV	13	24.600	8.140	0.007	0.843	0.721
POPD+PROT	13	25.243	8.782	0.005	0.835	0.706
POVGAP	14	29.533	9.546	0.004	0.796	0.618
ALL	**9**	**12.079**	**9.724**	**0.003**	**0.956**	**0.933**
GOV	14	35.711	15.724	<0.001	0.727	0.466
GDP	14	36.880	16.893	<0.001	0.714	0.437
POPG	14	39.336	19.349	<0.001	0.689	0.377
PROT	14	39.555	19.568	<0.001	0.687	0.371

Section 11. Boosted regression trees testing hypotheses using continuous variables

Using ranks to test our hypotheses could potentially mask trends given that the absolute differences between countries are not explicitly modelled. Despite the obvious advantages of ranks to provide more Gaussian-like error distributions, greater linearity, and equality of variances, we re-analysed the main dataset by first scaling and centring the component environmental metrics, and then taking their median value to generate a new environmental performance metric on a continuous scale. Next, we scaled and centred the the socio-economic predictor variables in the same manner, and then tested for relationships as we did for the ranked data.

However, the data violated several distributional assumptions necessary for structural equation models (Fig. S2-S4), so we instead applied a boosted-regression tree[24] approach implemented using the `dismo` R library and its function *gbm.step*[25], setting the error distribution family as Gaussian (scaling and centring all metrics ensured Gaussian behaviour), the bag fraction to 0.80, the learning rate to 0.0003, the tolerance to 0.0001, and the tree complexity to 2. The latter setting considers only first-order interactions and combines these effects if present into the relative influence scores (see below).

To assess the relative effect of each socio-economic variable on the scaled/centred, continuous-variable environmental performance metric, we calculated the boosted-regression tree metrics of relative influence[26]. Relative influence I is defined as the relative influences of the individual inputs x_j on the variation of the function $\hat{F}(x)$, the latter being the function that maps the explanatory variables x to the response variable y. For a collection of M decision (regression) trees $\{T_m\}_1^M$, the squared influence $\hat{I}^2$ is calculated as:

$$\hat{I}^2 = \frac{1}{M} \sum_{m=1}^{M} \hat{I}_j^2(T_m),$$

where $\hat{I}_j^2(T) = \sum_{t=1}^{J-1} \hat{\imath}_t^2(v_t = j)$, which is the summation over the nonterminal nodes t of the J-terminal node tree T, v_t is the splitting variable associated with node t, and $\hat{\imath}_t^2$ is the corresponding empirical improvement in squared error resulting from the split[27]. These squared influences then sum to 100 over all x explanatory parameters, which is analogous to the percentage of variation in the response variable y explained by each parameter. We assessed goodness-of-fit of each final tree structure using the cross-validation correlation coefficient β_{CV} (± its standard error calculated over all tree iterations).

Figure S2. Histograms of the scaled and centred composite variables comprising the median, continuous-variable environmental performance index.

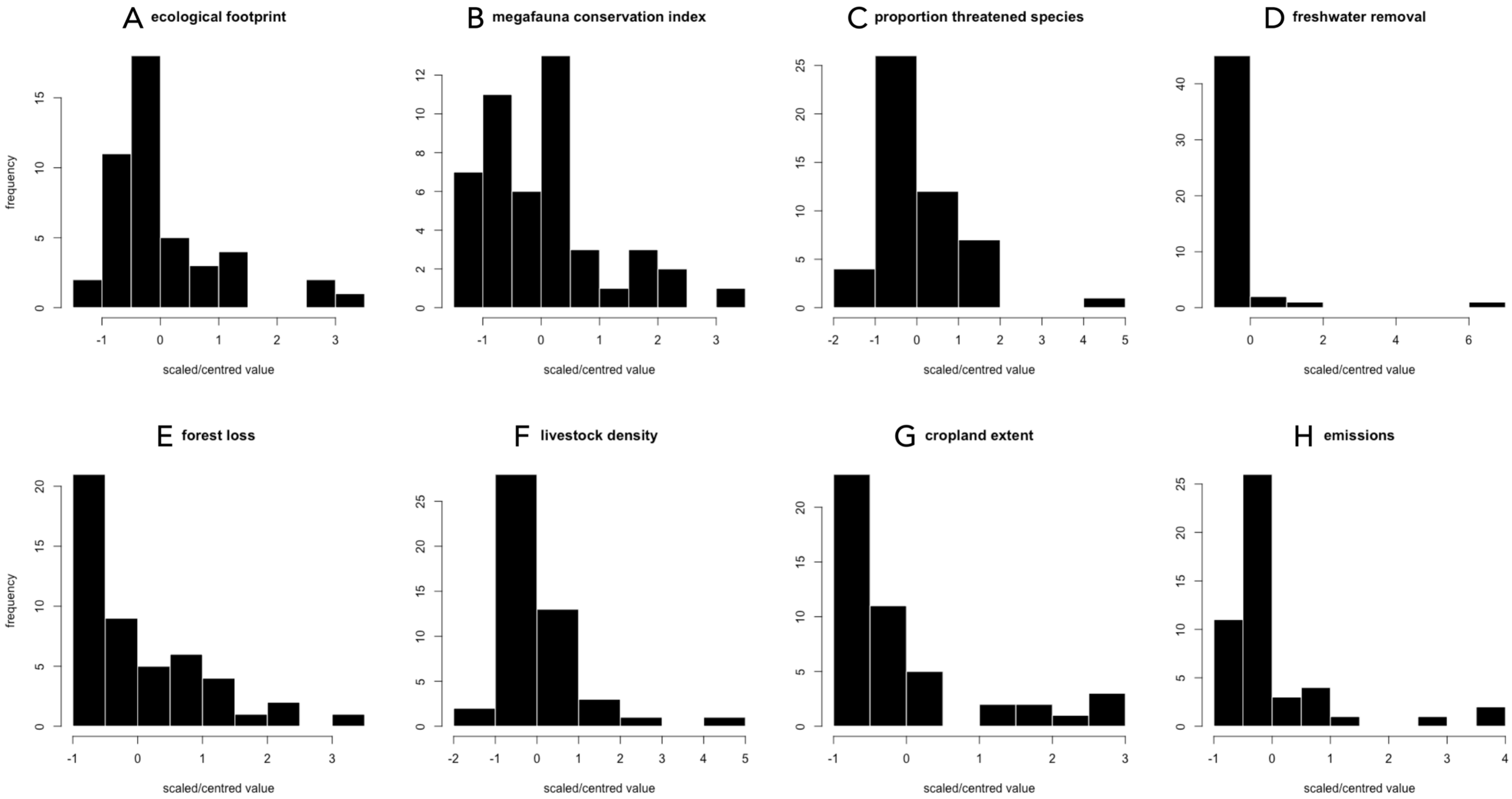

Figure S3. Histogram of the median environmental performance index based on the scaled and centred, continuous variables shown in Fig. S2.

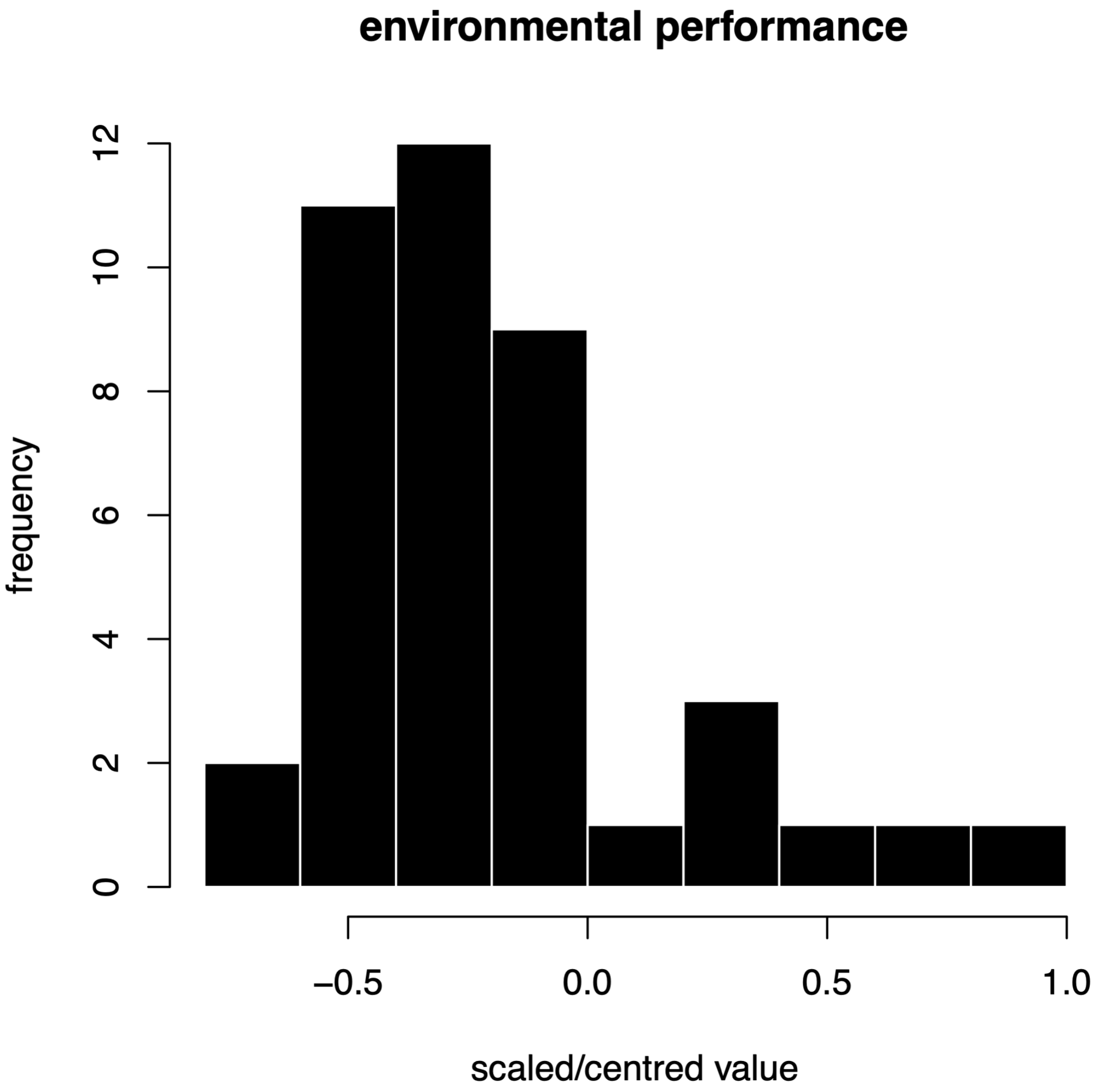

Figure S4. Histograms of the scaled and centred socio-economic predictor variables used to explain variation in the continuous-variable environmental performance index.

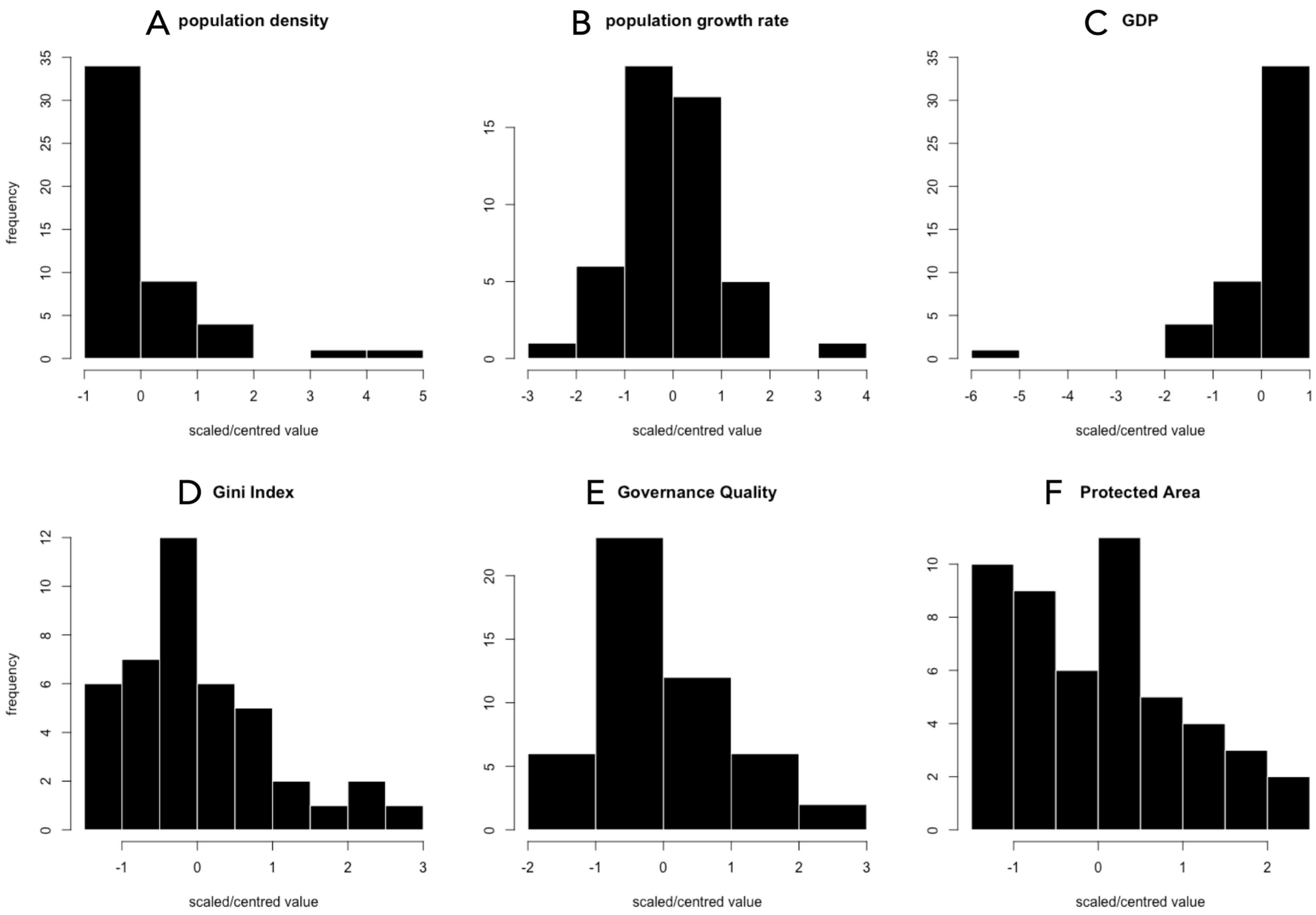

Figure S5. (**A**) The proportion of variance in child-health index explained by the socio-economic and environmental variables. Black bars indicate the relative contribution of each scaled and centred socio-economic variable to the variance in the scaled and centred, continuous median environmental performance index. The full dataset for the composite environmental performance index model used a total of 15200 trees (cross-validation correlation coefficient β_{CV} = 33.0 ± 19.6%). Predicted continuous environmental performance index as a function of variation in (**B**) population density (POPD), (**C**) population growth rate (POPG), (**D**) Gini wealth distribution index (GINI), and (**E**) per capita gross domestic product (GDP).

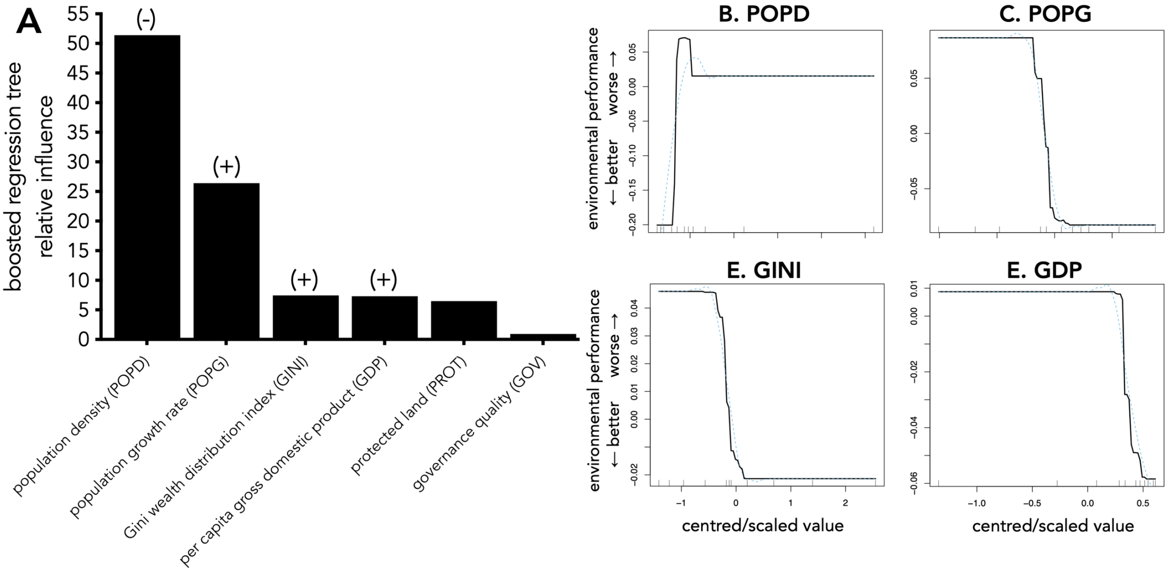

Section 12. Boosted regression tree fit for the relationship between environmental performance rank and per-capita GDP rank

We re-ran the rank-based structural equation model (see results in Table 4 of the main text) using a boosted regression tree approach (see Section 11 of the Supplementary Information Methods and Results for a description of the approach) to test whether the partialled (i.e., taking into account the variance explained by the other predictor variables — Table 4) effect of per-capita gross domestic product (GDP) rank on the environmental performance rank was 'U-shaped' (nonlinear) as is predicted according to the environmental Kuznets curve hypothesis[28]. In Fig. S6 below, there is no evidence for a U-shaped relationship, thus no support for the environmental Kuznets curve.

Figure S6. The predicted relationship between per-capita gross domestic product (GDP) rank and environmental performance rank according to a boosted regression tree approach. The dashed line is the smoothed fit. There is no evidence of a nonlinear (U-shaped) relationship between the two variables that would support the environmental Kuznets curve hypothesis.

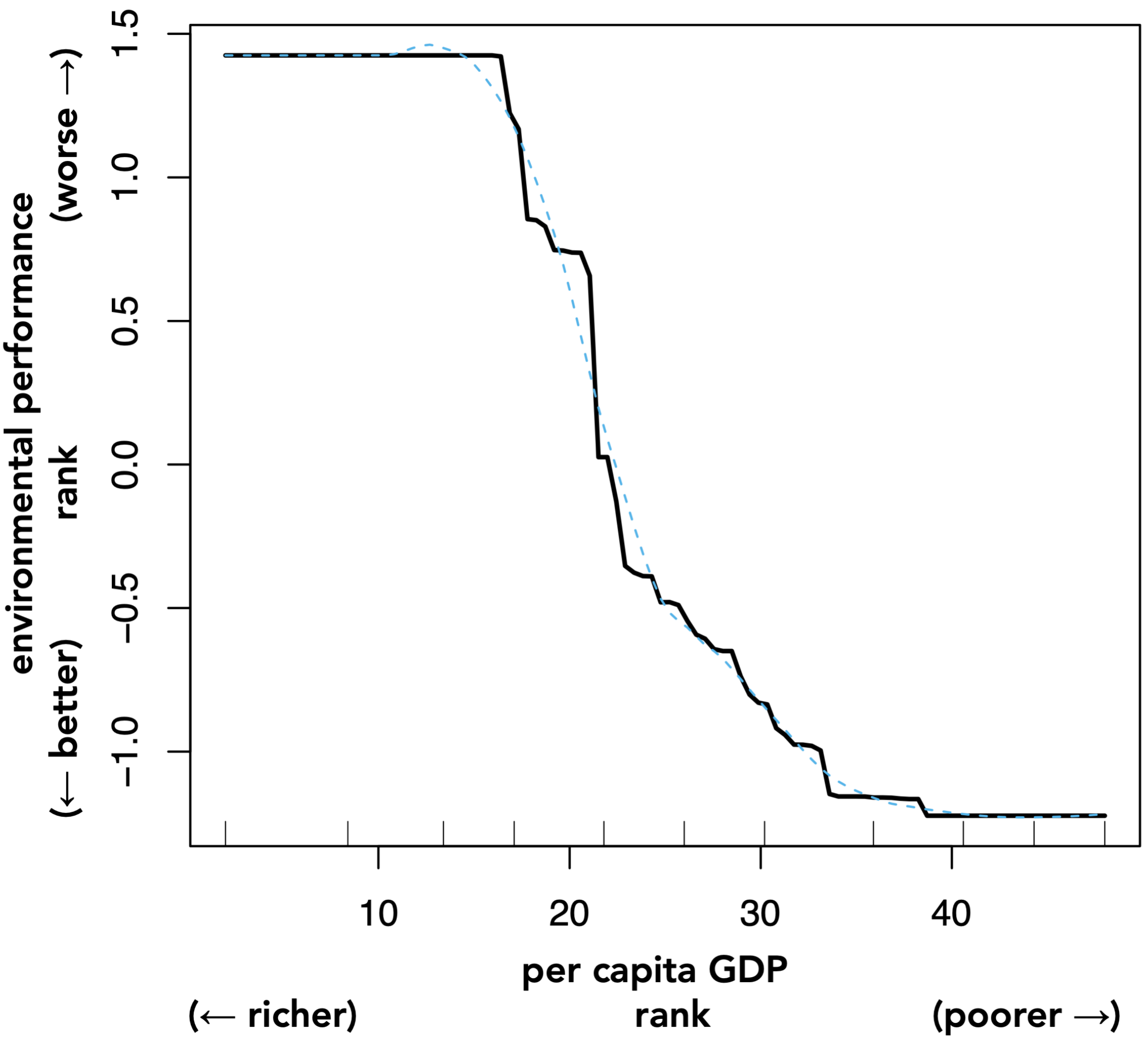

Supplementary Information References

1 Galli, A. *et al.* Questioning the Ecological Footprint. *Ecol. Indic.* **69**, 224-232, doi:10.1016/j.ecolind.2016.04.014 (2016).

2 Lindsey, P. A. *et al.* Relative efforts of countries to conserve world's megafauna. *Glob. Ecol. Conserv.* **10**, 243-252, doi:10.1016/j.gecco.2017.03.003 (2017).

3 Dudgeon, D. Prospects for sustaining freshwater biodiversity in the 21st century: linking ecosystem structure and function. *Curr. Opin. Env. Sustain.* **2**, 422-430, doi:10.1016/j.cosust.2010.09.001 (2010).

4 Davidson, N. C. How much wetland has the world lost? Long-term and recent trends in global wetland area. *Mar. Freshw. Res.* **65**, 934-941 (2014).

5 Maxwell, S. L., Fuller, R. A., Brooks, T. M. & Watson, J. E. M. The ravages of guns, nets and bulldozers. Nature 536: 146–145. *Nature* **536**, 143-145 (2016).

6 Achard, F. *et al.* Determination of deforestation rates of the world's humid tropical forests. *Science* **297**, 999-1002 (2002).

7 Hansen, M. C. *et al.* High-resolution global maps of 21st-Century forest cover change. *Science* **342**, 850-853, doi:10.1126/science.1244693 (2013).

8 Furukawa, T. *et al.* Forest harvest index: accounting for global gross forest cover loss of wood production and an application of trade analysis. *Glob. Ecol. Conserv.* **4**, 150-159, doi:10.1016/j.gecco.2015.06.011 (2015).

9 Thornton, P. K. Livestock production: recent trends, future prospects. *Phil. Trans. R. Soc. Lond. B* **365**, 2853-2867, doi:10.1098/rstb.2010.0134 (2010).

10 Pelletier, N. & Tyedmers, P. Forecasting potential global environmental costs of livestock production 2000–2050. *Proc. Natl. Acad. Sci. USA* **107**, 18371-18374 (2010).

11 Luck, G. W. A review of the relationships between human population density and biodiversity. *Biol. Rev.* **82**, 607-645, doi:10.1111/j.1469-185X.2007.00028.x (2007).

12 Bradshaw, C. J. A., Giam, X. & Sodhi, N. S. Evaluating the relative environmental impact of countries. *PLoS One* **5**, e10440, doi:10.1371/journal.pone.0010440 (2010).

13 Waldron, A. *et al.* Targeting global conservation funding to limit immediate biodiversity declines. *Proc. Natl. Acad. Sci. USA* **110**, 12144-12148, doi:10.1073/pnas.1221370110 (2014).

14 Smith, R. J., Muir, R. D. J., Walpole, M. J., Balmford, A. & Leader-Williams, N. Governance and the loss of biodiversity. *Nature* **426**, 67-70 (2003).

15 Laurance, W. F. Forest destruction in tropical Asia. *Current Sci* **93**, 1544-1550 (2007).

16 Mo Ibrahim Foundation. Ibrahim Index of African Governance. Detailed Methodology. (Mo Ibrahim Foundation, London, United Kingdom, 2016).

17 Bradshaw, C. J. A., Craigie, I. & Laurance, W. F. National emphasis on high-level protection reduces risk of biodiversity decline in tropical forest reserves. *Biol. Conserv.* **190**, 115-122, doi:10.1016/j.biocon.2015.05.019 (2015).

18 Bates, D., Maechler, M., Bolker, B. & Walker, S. lme4: linear mixed-effects models using Eigen and S4. *R package version 1.0-4*, http://CRAN.R-project.org/package=lme4 (2013).

19 Akaike, H. in *Proceedings of the Second International Symposium on Information Theory* (eds B. N. Petrov & F. Csaki) 267-281 (1973).

20 Link, W. A. & Barker, R. J. Model weights and the foundations of multimodel inference. *Ecology* **87**, 2626-2635, doi:10.1890/0012-9658(2006)87[2626:MWATFO]2.0.CO;2 (2006).

21 Nakagawa, S. & Schielzeth, H. A general and simple method for obtaining R^2 from generalized linear mixed-effects models. *Meth Ecol Evol* **4**, 133-142, doi:10.1111/j.2041-210x.2012.00261.x (2013).

22 McDonald, R. P. An index of goodeness of fit based on noncentrality. *J Classif* **6**, 97-103 (1989).

23 Bollen, K. A. A new incremental fit index for general structural equation models. *Soc Meth Res* **17**, 303-316 (1989).

24 Elith, J., Leathwick, J. R. & Hastie, T. A working guide to boosted regression trees. *J. Anim. Ecol.* **77**, 802-813, doi:10.1111/j.1365-2656.2008.01390.x (2008).

25 Hijmans, R. J., Phillips, S., Leathwick, J. & Elith, J. dismo: Species Distribution Modeling. R package version 1.1-4. CRAN.R-project.org/package=dismo. (2017).

26 Prowse, T. A. A. *et al.* An efficient protocol for the global sensitivity analysis of stochastic ecological models. *Ecosphere* **7**, e01238, doi:10.1002/ecs2.1238 (2016).

27 Friedman, J. H. Greedy function approximation: a gradient boosting machine. *Ann Stat* **29**, 1189-1232, doi:10.1214/aos/1013203451 (2001).

28 Stern, D. I., Common, M. S. & Barbier, E. B. Economic growth and environmental degradation: the environmental Kuznets curve and sustainable development. *World Dev* **24**, 1151-1160, doi:10.1016/0305-750X(96)00032-0 (1996).
